# Oncolytic Newcastle-disease-virus-mediated CD47 blockade in preclinical melanoma and pancreatic cancer models

**DOI:** 10.1016/j.omton.2025.201076

**Published:** 2025-11-01

**Authors:** Jacob G.E. Yates, Lily Chan, Alyssa E. Bogle, Elena S.B. Campbell, Arielle N. Gillies, Madison E. Hughes, Thomas M. McAusland, Leonardo Susta, Khalil Karimi, Samuel T. Workenhe, Sarah K. Wootton

**Affiliations:** 1 Department of Pathobiology, University of Guelph, Guelph, ON N1G 2W1, Canada; 2 Integrative Tumor Immunology and Immunotherapy, Institute Gustave Roussy, 94800 Villejuif, France

**Keywords:** MT: Regular Issue, NDV, oncolytic virus, CD47, cancer immunotherapy, immune checkpoint blockade, immune checkpoint inhibitors

## Abstract

Newcastle disease virus (NDV) exhibits strong immunostimulatory activity and a favorable safety profile, but its moderate monotherapy efficacy may be improved through combination therapies. Although NDV selectively infects and lyses tumor cells, viral infection unintentionally elevates tumor-cell CD47 expression—a “don’t-eat-me” signal that could mask the full immunostimulatory potential of NDV. To offset this adverse host response, we engineered NDVs that expressed an anti-CD47 antibody or SIRPα-Fc immunoadhesin to pair direct oncolysis with local CD47 blockade to amplify SIRPα^+^ phagocytic-cell-mediated clearance of tumor cells and downstream antitumor immunity, as well as circumvent dose-related toxicities associated with systemic administration of CD47-blocking agents. NDV-mediated CD47 blockade decreased intratumoral CD47 expression and increased CD8 T cell activation, with no significant changes in antigen-presenting cell activation or phagocytosis. NDV-mediated CD47 blockade did not improve animal survival in B16-F10 melanoma nor KPC pancreatic ductal adenocarcinoma (PDAC), but greater incidence of tumor clearance resulting in immunological memory was observed in PDAC compared to B16-F10 tumor-bearing mice. Although NDV-mediated CD47 blockade resulted in increased numbers of PD-1^+^ CD8 T cells, synergy between NDV, CD47, and PD-L1 blockade was limited. Together these data highlight the importance of considering tumor-intrinsic factors when combining cancer immunotherapies for improved outcomes.

## Graphical abstract

**Figure:**
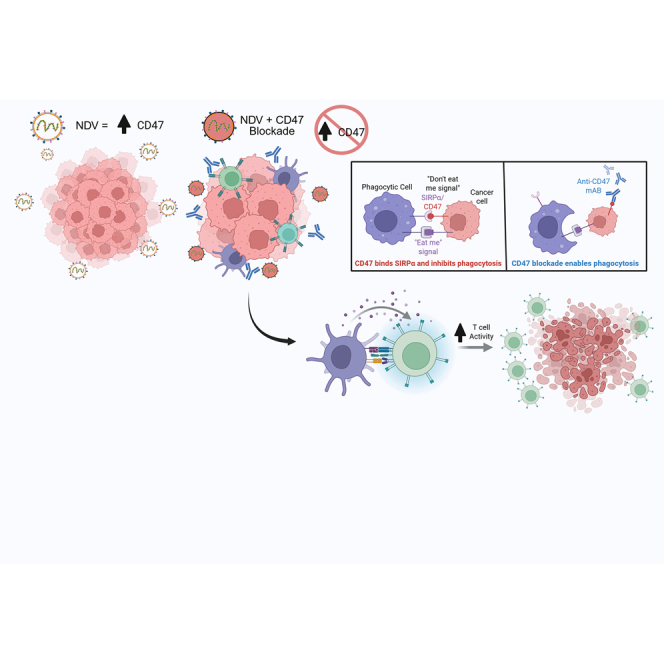

## Teaser

Oncolytic viruses, like Newcastle disease virus (NDV), support anti-tumor immunity. Here, Wootton and colleagues engineered NDV for targeted and localized CD47 blockade in murine melanoma and pancreatic ductal adenocarcinoma. Benefits of this novel combination immunotherapy varied between tumor type, highlighting the importance of identifying tumor markers that indicate immunotherapy responsiveness.

## Introduction

Newcastle disease virus (NDV) has a long-standing history as an oncolytic virus (OV) and more recently as a vaccine vector.^1^^,^^2^^,^^3^^,^^4^ As an oncolytic virus, NDV selectively infects and replicates in cancer cells, producing a pro-inflammatory tumor microenvironment and activating anti-cancer immunity.^5^^,^^6^^,^^7^^,^^8^^,^^9^^,^^10^ Clinical studies administering NDV as a monotherapy have demonstrated its favorable safety profile but limited therapeutic efficacy, underscoring the need for strategies that enhance its potency as a cancer immunotherapeutic agent.^11^^,^^12^^,^^13^^,^^14^ This is similar to other OVs, like herpes simplex virus (HSV), adenovirus, and vaccinia virus, with HSV encoding granulocyte-macrophage colony-stimulating factor (GM-CSF) having received Food and Drug Administration (FDA) approval for use as a cancer immunotherapy.^15^^,^^16^^,^^17^ NDVs’ natural oncolytic abilities, paired with its strong safety profiles and immunostimulatory capabilities, make them an attractive viral vector for vaccination and cancer immunotherapy.

NDVs can be engineered to express therapeutic payloads following viral infection.^6^^,^^9^^,^^10^^,^^15^^,^^16^^,^^18^^,^^19^ This has enabled localized delivery of immune checkpoint inhibitors (ICIs) and cytokines, which have improved the efficacy of NDVs as a cancer immunotherapy.^6^^,^^9^^,^^10^^,^^15^^,^^16^^,^^18^^,^^19^ Most recently, NDV encoding GM-CSF, in combination with anti-PD-L1 treatment, was evaluated in a phase I clinical trial where improved partial responses were observed.^12^

When NDV has been combined with ICIs, it has predominantly been in the context of innate:adaptive immune signaling.^6^^,^^10^^,^^19^ This includes PD-L1:PD-1 and CD80/86:CTLA-4, which act to block inhibitory signaling between tumor cells and T cells and antigen-presenting cells (APCs) and T cells, respectively.^20^^,^^21^^,^^22^^,^^23^ NDV has yet to be evaluated in combination with an ICI that targets a tumor:innate immune signaling pathway, such as CD47:SIRPα. CD47 is ubiquitously expressed, and its regulation is linked to inflammation.^24^^,^^25^ The interaction between CD47 and SIRPα on phagocytic cells prevents phagocytosis. CD47 is often upregulated on tumor cells as a means of survival and immune evasion.^24^^,^^25^ Therefore, blockade of CD47 supports the development of anti-tumor immunity by increasing phagocytosis of tumor cells and the presentation of antigen to T cells.^24^^,^^26^ When administered systemically, CD47-blocking agents are limited by on-target, off-tumor specificity due to the ubiquitous expression of CD47, especially on red blood cells, which has resulted in adverse side effects such as anemia.^24^^,^^27^^,^^28^ This issue has been addressed to some extent by using loading doses prior to higher therapeutic doses.^24^^,^^27^^,^^28^

We rationalized that engineering NDV to express an anti-CD47 monoclonal antibody or a SIRPα-Fc immunoadhesin would enable localized delivery of CD47-blocking agents to the tumor microenvironment, thereby enhancing therapeutic specificity while minimizing on-target, off-tumor effects. This novel combination immunotherapy of NDV and CD47 ICIs leverages the induction of a proinflammatory tumor microenvironment and recruitment of anti-tumor immune cells by NDV, with increased phagocytosis and antigen presentation mediated by CD47 blockade. Shown here are the implications of NDV-mediated CD47 blockade in murine models of melanoma and pancreatic ductal adenocarcinoma. Recombinant NDVs (rNDVs) decreased intratumoral CD47 expression and increased the proportion of PD-1^+^ T cells while no change to APCs was directly observed with B16-F10-bearing mice. Survival benefits varied between tumor types, with KPC-bearing mice exhibiting greater incidences of complete remission and a resistance to re-challenge. This contrasted the B16-F10 model, where an incidence of complete remission was only observed following the addition of PD-L1 blockade.

## Results

### NDV infection leads to CD47 upregulation on tumor cells

**Figure 1:**
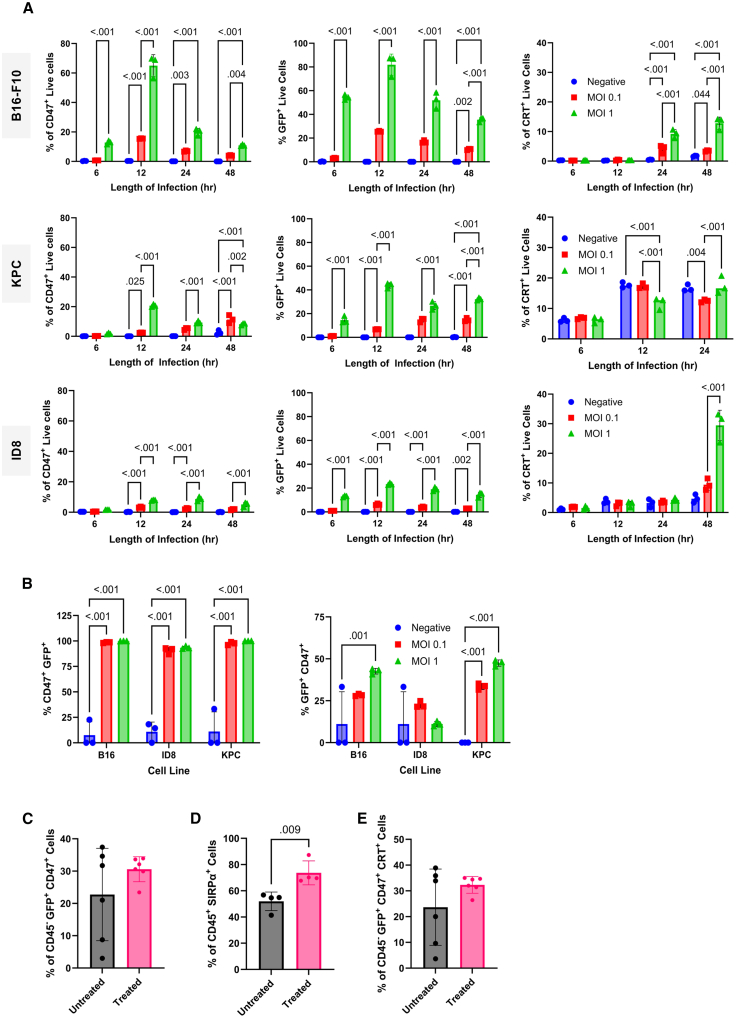
Impact of NDV infection on CD47 expression in murine tumor cells (A) Murine melanoma (B16-F10), pancreatic (KPC), or ovarian (ID8) cancer cells were left uninfected or infected at a multiplicity of infection of 1 or 0.1 with mesogenic NDV-GFP. Cells were harvested at 6, 12, 24, and 48 h post-infection to assess changes in CD47 expression, virus-mediated expression of GFP, and surface expression of calreticulin by flow cytometry (*n* = 3 independent experiments). (B) Co-expression of CD47 and GFP following NDV-GFP infection (*n* = 3 independent experiments). (C–E) C57BL/6 mice bearing intradermal B16-F10-GFP tumors (∼5 × 5 mm) (*n* = 4) were treated intratumorally with 1 × 10^8^ PFU of NDV-Luciferase. Twelve hours later, tumors were harvested to evaluate tumor cell expression of CD47 on tumor cells (GFP+, CD47+) (C), SIRPα on CD45+ cells (D), and surface calreticulin on tumor cells (GFP+, CRT+) (E). Significance was assessed in (A) and (B) using two-way ANOVA with Tukey’s multiple comparisons test and unpaired parametric *t* tests with Tukey’s multiple comparison in (C). Shown are means ± SD.

CD47 overexpression is a well-established immune evasion strategy in many malignancies where it inhibits macrophage phagocytosis.^29^^,^^30^ Moreover, since oncolytic viruses can trigger compensatory upregulation of immunosuppressive proteins, as demonstrated by NDV-induced PD-L1 expression on tumor cells,^6^ we sought to characterize CD47 expression levels in murine KPC and B16-F10 cancer cells and investigate whether NDV infection modulates CD47 expression. To evaluate the impact of NDV infection on the expression of anti-phagocytic CD47 and the pro-phagocytic surface calreticulin (CRT), we infected three murine tumor cell lines with GFP expressing NDV. B16-F10 melanoma, KPC pancreatic ductal adenocarcinoma, and ID8 ovarian cancer cells were infected at an MOI of 0.1 or 1, and surface expression of CD47 and CRT was evaluated at 6, 12, 24, and 48 h post-infection (Figure 1A). Translocation of CRT from the endoplasmic reticulum to the plasma membrane serves as an “eat-me” signal to phagocytic cells. Therefore, a simultaneous increase in pro-phagocytic surface CRT and decrease in CD47 would suggest increased tumor cell phagocytosis. Expression of CD47 was rapidly upregulated in all the three tumor cell lines evaluated, with peak expression observed 12 h post-infection (Figure 1A). The number of CD47^+^ cells increased in a dose-dependent manner, with a larger percentage of tumor cells with elevated CD47 expression when a higher MOI of NDV was used. This suggests that NDV infection leads to the upregulation of CD47 expression.

Of the three cancer cell lines examined, B16-F10 cells were most susceptible to NDV-GFP infection, as evidenced by the highest percentage of GFP-positive cells, and this directly correlated with CD47 expression (Figure 1A). KPC and ID8 cancer cells were less susceptible to NDV infection as evidenced by lower numbers of GFP-positive cells and correspondingly lower numbers of CD47^+^ cells. However, this trend did not appear to hold true with respect to the surface expression of CRT. In B16-F10 and ID8 cell lines, it took longer to observe an increase in surface CRT, peaking at 48 h post-NDV-GFP infection (Figure 1A). Increases in CRT appeared to be dose dependent, with a higher MOI resulting in greater CRT expression. However, this increase was not linked to NDV’s ability to replicate in these cell lines, as ID8 cells exhibited the largest CRT^+^ population but the smallest number of GFP^+^ cells. Interestingly, KPC cells exhibited higher basal expression of surface CRT, which did not appear to be modulated by NDV infection. Similar trends were observed when NDV-GFP was used in SKMEL-28 and UACC-62 human melanomas, with peak CD47 expression occurring in a dose-dependent manner (Figure S1). To further investigate the relationship between NDV infection and the upregulation of CD47, co-expression of both GFP and CD47 was evaluated (Figure 1B). Almost all CD47^+^ cells were GFP^+^ but not all GFP^+^ cells were CD47^+^, indicating that most infected cells upregulate CD47 (Figure 1B).

As B16-F10 exhibited the largest increase in CD47 and expression of GFP following NDV-GFP infection, we sought to investigate whether this trend would be observed *in vivo*. As peak CD47 expression occurred 12 h post-infection *in vitro*, tumors were harvested 12 h post-intratumoral administration of 1 × 10^8^ plaque-forming units (PFU) of NDV-expressing luciferase (NDV-Luc), and CD47 and GFP expression levels were assessed by flow cytometry. As was observed *in vitro*, NDV infection led to an increase in the number of GFP^+^ CD47^+^, CD45^+^ SIRPα^+^, and CRT^+^ GFP^+^ tumor cells (Figures 1C–1E). Untreated tumors exhibited greater variability in their expression of CD47 and surface CRT, with the impact of NDV infection less apparent than during *in vitro* infections (Figures 1C and 1D). However, NDV infection resulted in a significant recruitment of CD45^+^ SIRPα^+^ cells to the tumor (Figure 1E).

### rNDVs express functional CD47-blocking agents

**Figure 2:**
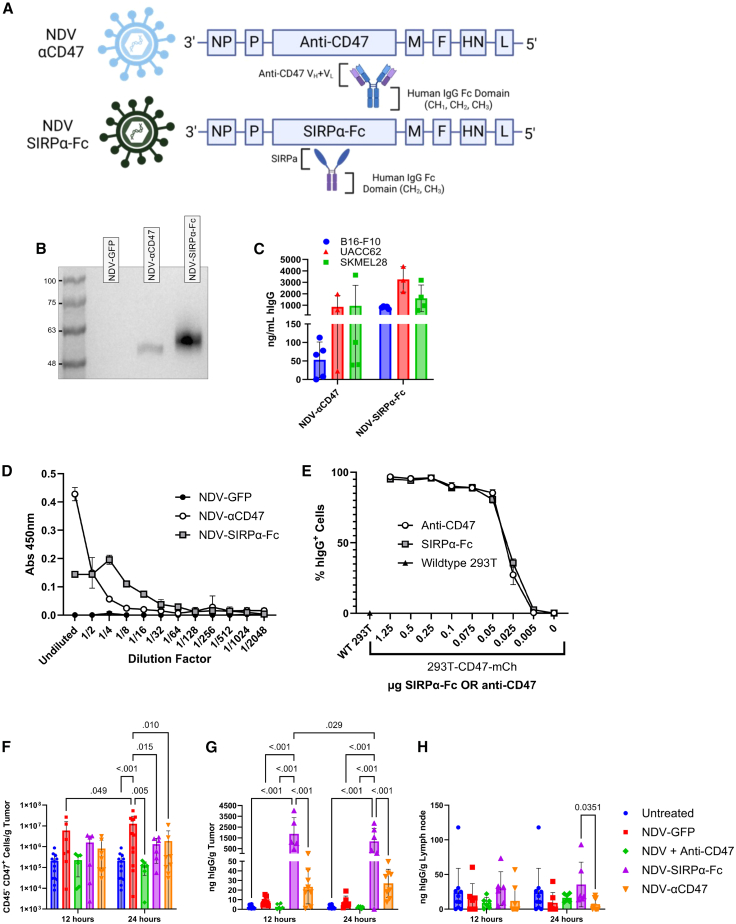
Engineering and functional analysis of NDVs for tumor-targeted CD47 blockade (A) Genomic design of recombinant NDV vectors. (B) Western blot of allantoic fluid from eggs infected with NDV-GFP, NDV-αCD47, or NDV-SIRPα-Fc. Equal volumes of allantoic fluid from SPF embryonated chicken eggs infected with NDV-GFP (1), NDV-αCD47 (2), or NDV-SIRPα-Fc (3). (C) Human IgG levels in supernatants of B16-F10 (*n* = 5), UACC-62 (*n* = 3), or SKMEL-28 (*n* = 4) cells infected with NDV-GFP, NDV-αCD47, or NDV-SIRPα-Fc (MOI = 1) quantified by hIgG ELISA after 48 h. Values normalized to NDV-GFP. (D) Functional binding ELISA of 2-fold serially diluted NDV-GFP, NDV-CD47, or NDV-SIRP-Fc allantoic fluid on wells coated with murine CD47 and detected with anti-human-IgG HRP conjugate. Shown are three biological replicates at each dilution. (E) HEK293T cells engineered to overexpress mCherry and murine CD47 were stained with purified anti-CD47 or SIRPα-Fc prior to staining with anti-hIgG-PECy7 and flow cytometric analysis (three biological replicates are shown for each dilution). (F–H) C57BL/6 mice with intradermal B16-F10 tumors (∼5 × 5mm) were either left untreated (*n* = 12) or treated intratumorally with 1 × 10^8^ PFU of the indicated NDV vector (*n* = 6–12), 50 μg of an anti-CD47 mAb (*n* = 5) (every 3 days), or a combined therapy. At 12 and 24 h post-treatment, tumors were evaluated for CD47 expression (CD45−) (F). Presence of hIgG was quantified in tumors (G) and tumor draining lymph nodes (H) by hIgG ELISA (*n* = 5–12). Shown are means ± SD. Significance was assessed by two-way ANOVA with Tukey’s multiple comparisons.

Having shown the dose-dependent relationship between NDV infection and increased CD47 expression, two rNDV vectors were engineered to block CD47/SIRPa signaling (Figure 2A). NDV-αCD47 encodes an anti-mouse CD47 monoclonal antibody with a human immunoglobulin G1 (IgG1) (hIgG) Fc domain. NDV-SIRPα-Fc encodes a SIRPα-Fc immunoadhesin that contains the extracellular portion of SIRPα fused to the CH2-CH3 domains of the human IgG1 Fc domain (Figure 2A). The ability of these rNDVs to express the desired transgenes in allantoic fluid was evaluated by western blots. As shown in Figure 2B, anti-CD47 and SIRPα-Fc were detected at the estimated molecular weights of ∼50 and ∼58 kDa, respectively, in the allantoic fluid of NDV-αCD47- and NDV-SIRPα-Fc-infected specified pathogen-free (SPF) embryonated chicken eggs. Transgene expression was then verified in tumor cell lines using a quantitative anti-hIgG enzyme-linked immunosorbent assay (ELISA). Murine B16-F10 and human UACC-62 and SKMEL-28 melanomas were infected with NDV-αCD47 and NDV-SIRPα-Fc at an MOI of 1 for 48 h after which supernatants were collected to measure hIgG by ELISA (Figure 2C). For both recombinant viruses, transgene expression was higher in the human melanoma cell lines than the B16F-10 murine cell line. SIRPα-Fc expression from NDV was higher than that of αCD47 in all three cell lines tested. Next, the ability of the anti-CD47 antibody and SIRPα-Fc immunoadhesin produced by these rNDVs to bind to CD47 was evaluated using two different assays. Employing a CD47 binding ELISA, allantoic fluid from SPF embryonated chicken eggs inoculated with NDV-αCD47 or NDV-SIRPα-Fc, bound to CD47, whereas allantoic fluid harvested from NDV-GFP-infected eggs did not (Figure 2D). Similar results were observed when different concentrations of purified anti-CD47 and SIRPα-Fc were used to detect the expression of CD47 on CD47-overexpressing HEK293T cells (Figure 2E).

Changes to viral fitness were assessed by multi-step growth curve and resazurin assays, as a surrogate for cell viability in B16-F10, UACC-62, and SKMEL-28 melanoma cell lines (Figure S2; Tables S1–S6). Both rNDVs exhibited a fitness similar to a control NDV expressing GFP, with NDV-αCD47 trending toward a lower replicative ability (Figure S2). Consistent with the findings presented in Figure 1B, human melanoma cell lines appear more sensitive to NDV killing, as evidenced by fewer metabolically active cells.^9^

Having confirmed *in vitro* binding of our CD47-blocking agents, we next characterized the impact of intratumoral administration of these rNDVs *in vivo* in a B16-F10 melanoma model since we observed the highest level of CD47 upregulation and transgene expression following NDV infection in B16-F10 cells (Figure 1A). Approximately 7–10 days after intradermally implanting 2 × 10^5^ B16-F10 cells, 1 × 10^8^ PFU of NDV-αCD47, NDV-SIRPα-Fc, NDV-GFP, or NDV-Luciferase in combination with 50 μg of anti-CD47 given independently was administered. At 12 and 24 h post-administration, tumors receiving treatments containing CD47-blocking agents exhibited less CD47 expression (Figure 2F). hIgG was detected only in the tumor tissue but not the tumor-draining lymph node (tdLN) and serum, suggesting localized expression of NDV vectorized CD47-blocking agents (Figures 2G, 2H, andS3). Taken together, these findings demonstrate that NDV can be engineered to successfully express an anti-CD47 antibody or SIRPα-Fc immunoadhesin that binds CD47, confirmed both *in vitro* and *in vivo*. Moreover, these vectorized CD47 blocking agents are localized within the tumor microenvironment.

### Vectorized CD47 blockade does not enhance NDV-mediated survival in B16-F10 melanoma

**Figure 3:**
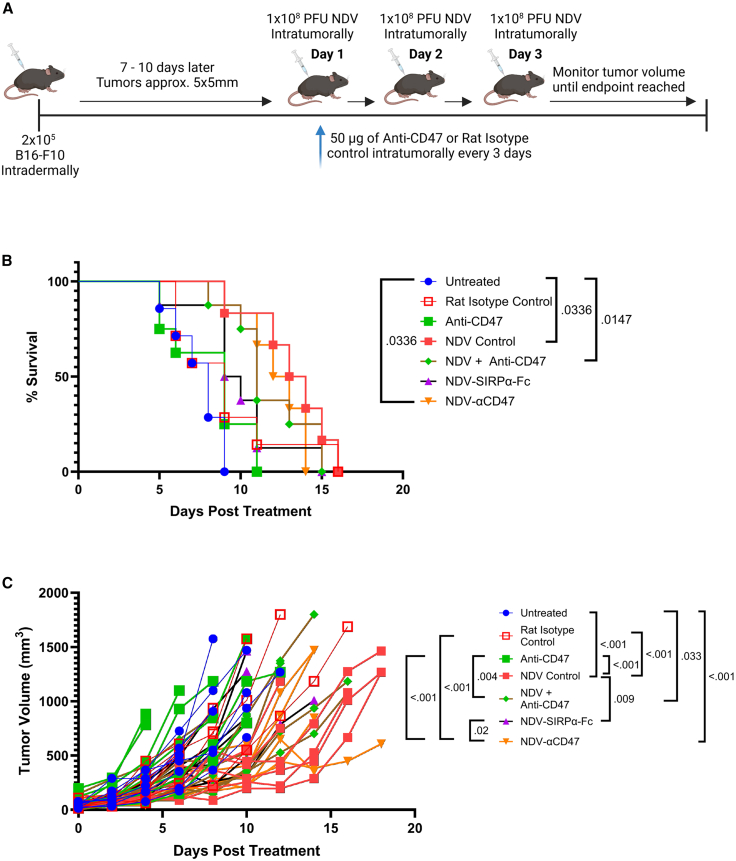
Assessment of NDV-mediated CD47 blockade in B16-F10 melanoma (A) Schematic presentation of B16-F10 treatment strategy. C57BL/6 mice (*n* = 6–8 per group) bearing B16-F10 tumors (∼5 × 5mm) were treated intratumorally with the indicated NDV vector (1 × 10^8^ PFU daily, 3 days), anti-CD47 or isotype control mAb (50 μg every 3 days), or a combined therapy. (B) Kaplan-Meier survival curve. (C) Tumor volumes measured every other day using [(L)×(W)^2^]/2. Significance was assessed by log rank test with Bonferroni correction and Cox proportional hazards regression (B) or two-way ANOVA with Tukey’s multiple comparison (C).

Having engineered NDV vectors that mediate localized CD47 blockade, we sought to examine whether these rNDVs mediate a survival benefit in the B16-F10 melanoma model, as our *in vitro* results indicated B16-F10 melanoma supported the most transgene production and had the highest increase in CD47 expression (Figure 1A). Groups of B16-F10-bearing mice were administered either PBS (*n* = 7), 1 × 10^8^ PFU NDV-Luc (*n* = 6), NDV-αCD47 (*n* = 6), NDV-SIRPα-Fc (*n* = 8), NDV-Luc in combination with 50 μg anti-CD47 given independently (*n* = 8), 50 μg anti-CD47 antibody alone (*n* = 8), or 50 μg of an isotype control alone (*n* = 7) (Figure 3A), and tumor volume was monitored over time. Intratumoral administration of anti-CD47 antibody alone did not mediate a significant survival benefit relative to PBS-treated mice (Figure 3B) nor did it delay tumor growth (Figure 3C). Kaplan-Meier analysis of all treatments containing NDV, other than NDV-SIRPα-Fc, significantly extended survival compared to PBS-treated mice and significantly delayed tumor growth to varying degrees (Figures 3B and 3C). Cox proportional hazards regression also indicated that NDV alone and NDV-vectorized CD47 blockade mediated significantly reduced death hazard relative to untreated mice (Figure S4). NDV, NDV-αCD47, and NDV in combination with recombinant anti-CD47 given independently also significantly reduced the hazard of death relative to anti-CD47 as a monotherapy (Figure S4).

### NDV and CD47 blockade improves T cell activation in B16-F10 melanoma

**Figure 4:**
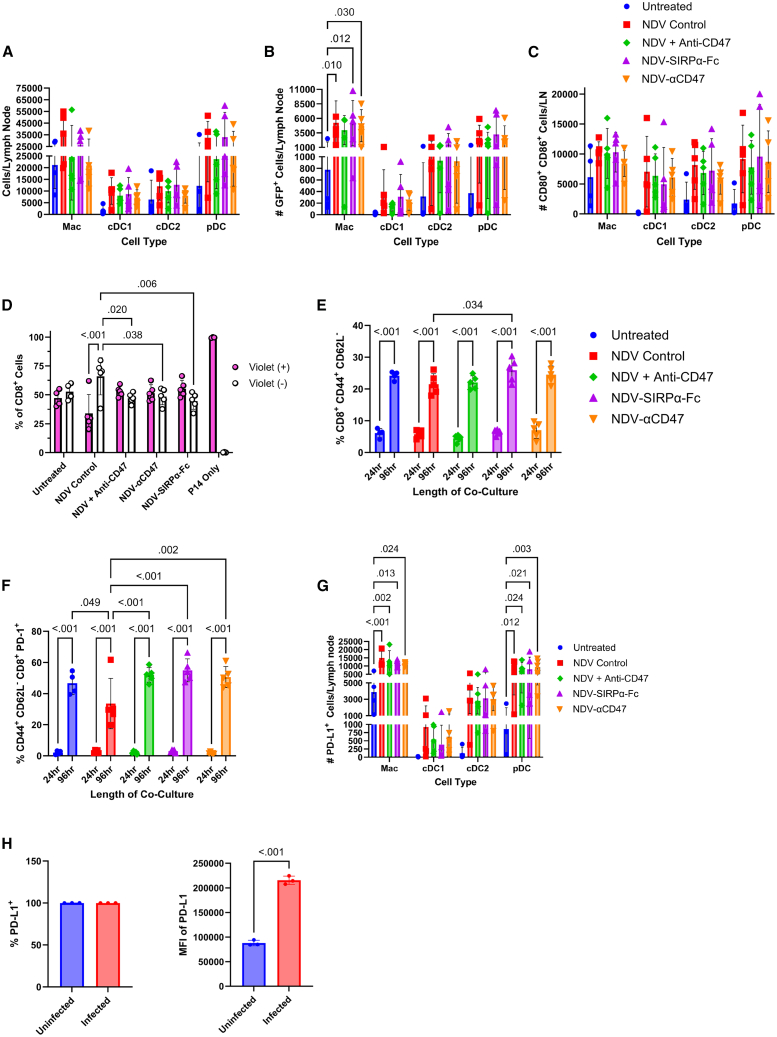
Evaluation of APC and CD8 T cell responses to NDV-mediated CD47 blockade in B16-F10 melanoma (A–C) Mice (*n* = 5) intradermally implanted with B16-F10-zsGreen-GP33 tumors were treated with NDV plus CD47 blockade and 24 h post-treatment, and tdLNs were harvested and processed into single-cell suspensions for immunophenotyping. (D and E) Bulk lymphocytes from tdLNs of B16-F10-zsGreen-GP33-tumor-bearing mice were co-cultured with bulk CellTraceViolet-stained P14 splenocytes (1:3) for 24 or 96 h prior to immunophenotyping (*n* = 4 technical replicates). (F and G) C57BL/6 mice (*n* = 5) bearing intradermal B16-F10-zsGreen-GP33 tumors (∼5 × 5mm) were treated intratumorally with 1 × 10^8^ PFU of an NDV vector or NDV plus 50 μg anti-CD47 mAb. Twenty-four hours later, tumor-draining lymph nodes were harvested for flow cytometric analysis (F), or bulk lymphocytes were co-cultured with bulk CellTraceViolet-stained P14 splenocytes (1:3) for 24 or 96 h, then stained for flow cytometric analysis (G). (H) B16-F10 cells were left uninfected or infected with NDV-GFP at an MOI of 1 for 18 h prior to flow cytometric analysis (*n* = 3 independent experiments). Shown are means ± SD. Significance was assessed by two-way ANOVA with Tukey’s multiple comparisons (A–G) and unpaired parametric *t* test (H).

We next investigated whether CD47 blockade was eliciting the desired effect on different APC subsets, type I and type II conventional dendritic cells (cDC1 and cDC2), plasmacytoid dendritic cells (pDCs), and macrophages using recombinant B16-F10 tumor cells engineered to express zsGreen and the lymphocytic choriomeningitis virus (LCMV) glycoprotein (GP) epitope GP33. Mice intradermally implanted with B16-F10-zsGreen-GP33 tumors were treated with NDV and CD47 blockade and tdLNs harvested 24 h post-treatment, as this is when most APCs are found in the tdLN (Figure S5). There was no significant change in the number of APCs infiltrating the tdLN relative to NDV alone (Figure 4A). Furthermore, there was no significant change in tumor cell phagocytosis or the expression of co-stimulatory markers CD80 and CD86 when compared to NDV alone (Figures 4B and 4C). Since CD47 blockade has been described to increase antigen presentation and greater T cell activity, we investigated this by assessing effector CD8 T cell proliferation, quantity, and programmed cell death protein 1 (PD-1) expression. When bulk lymphocytes from tdLNs of B16-F10-zsGreen-GP33-tumor-bearing mice were co-cultured with bulk splenocytes from P14 mice (mice bearing naive CD8 T cells with specificity for GP33) for 4 days, fewer CD8 T cells had undergone proliferation, as determined by the absence of CellTraceViolet (Figure 4D). After co-culturing bulk lymphocytes with bulk P14 splenocytes for 4 days, only lymphocytes from mice treated with NDV-SIRPα-Fc exhibited a significantly greater CD8 effector T cell subset (CD44^+^ and CD62L^−^) (Figure 4E).^31^^,^^32^ The remaining treatments, NDV-Luc, anti-CD47, NDV-αCD47, or NDV in combination with recombinant anti-CD47 given independently, did not significantly alter the proportion of the effector CD8 T cell population. However, when lymphocytes from the tdLNs of treated mice were co-cultured with splenocytes from P14 mice, those that received NDV and CD47 blockade exhibited significantly more PD-1 compared to mice that received only NDV (Figure 4F). This prompted the investigation of PD-L1 expression in lymphocytes within the tdLN and B16-F10 tumors. Lymphocytes from NDV- and CD47-treated tdLNs exhibited PD-L1 expression similar to that of mice receiving only NDV (Figure 4G). Furthermore, B16-F10 cells expressed high levels of PD-L1, which was increased following NDV infection (Figure 4H). In summary, CD47 blockade with NDV failed to enhance APC function or CD8 T cell proliferation but increased PD-1 on CD8 T cells alongside high tumor PD-L1.

### Dual CD47/PD-L1 blockade improves survival and can induce complete remission in the B16-F10 melanoma model

**Figure 5:**
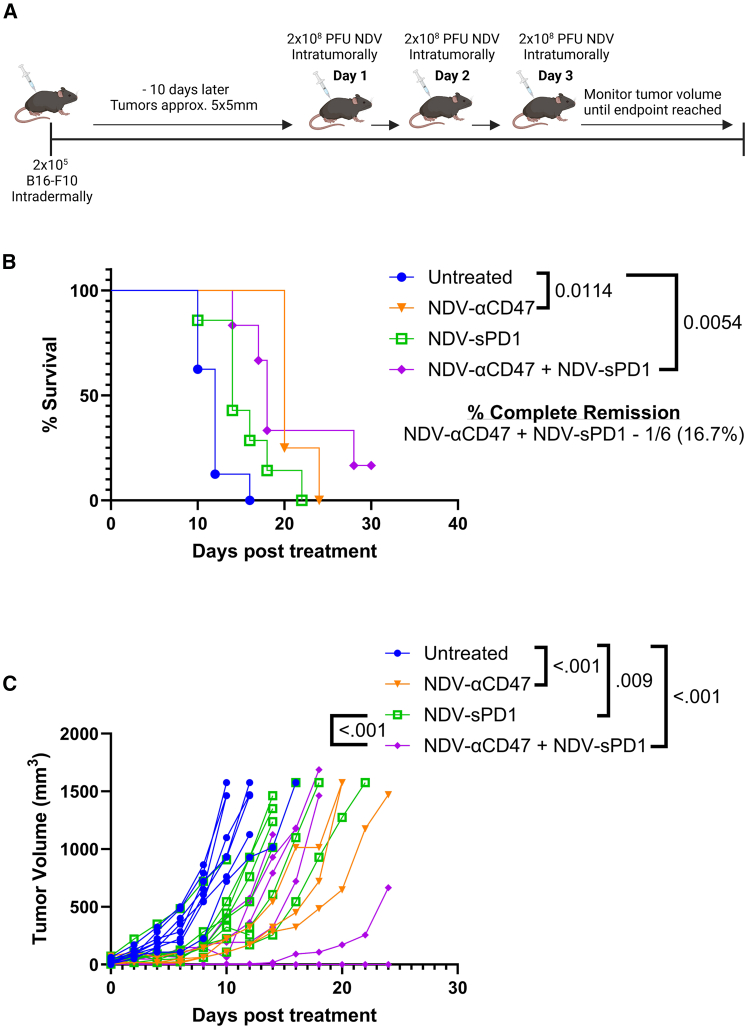
Evaluation of NDV-mediated CD47 and PD-L1 blockade in B16-F10 melanoma (A) Schematic outlining of the combined NDV-vectorized CD47 and NDV-vectorized PD-L1 blockade model. (B, C) C57BL/6 mice (*n* = 6–8) with intradermal B16-F10 tumors (∼5 × 5 mm) were treated daily for 3 days with 2 × 10^8^ PFU NDV-Luciferase or NDV-αCD47 plus NDV-Luciferase or NDV-sPD1 plus NDV-Luciferase or NDV-αCD47 plus NDV-sPD1 (1 × 10^8^ PFU each NDV, 2 × 10^8^ PFU total). (B) Kaplan-Meier survival curve. (C) Record of daily tumor growth [(L)×(W)^2^]/2. Shown are mean ± SD. Significance was assessed by log rank test with Bonferroni correction and Cox proportional hazards regression (B) or two-way ANOVA with Tukey’s multiple comparisons (C).

Given the increase in PD-1 expression observed on CD8^+^ T cells and the high levels of PD-L1 expression on B16-F10 tumors, we next evaluated whether combining PD-L1 blockade and CD47 blockade provided any synergy, as has been seen previously.^33^^,^^34^ B16-F10-bearing mice received an rNDV vector encoding an anti-CD47 monoclonal antibody (mAb) (NDV-αCD47), a soluble PD-1 that blocks PD-L1 (NDV-sPD1), or both vectors (Figure 5A). Following Kaplan-Meier analysis, NDV-αCD47 and NDV-αCD47 + NDV-sPD1 provided a significant survival benefit compared to untreated mice. However, combined PD-L1 and CD47 blockade did not significantly improve upon either NDV-αCD47 or NDV-sPD1 treatment alone, and similar increases in tumor volumes were observed (Figures 5B and 5C), although one mouse in the combined CD47 and PD-L1 blockade group experienced complete remission (CR) (Figure 5B). Cox proportional hazard analysis identified all treatments as significantly improving survival, with combined CD47 and PD-L1 blockade having the greatest impact (Figure S6). Additionally, combined CD47 + PD-L1 blockade significantly reduced the hazard of death compared to PD-L1 blockade alone (Figure S6). Overall, the combination of NDV with CD47 blockade reduced the intratumoral level of CD47 but did not increase tumor antigen phagocytosis by APCs or the proliferation of effector CD8 T cells. However, CD8 T cells exhibit increased PD-1 expression following CD47 blockade, which when combined with PD-L1 blockade, increased the incidence of CR (Figure 5B).

### NDV plus CD47 blockade improves immunological memory in KPC PDAC

**Figure 6:**
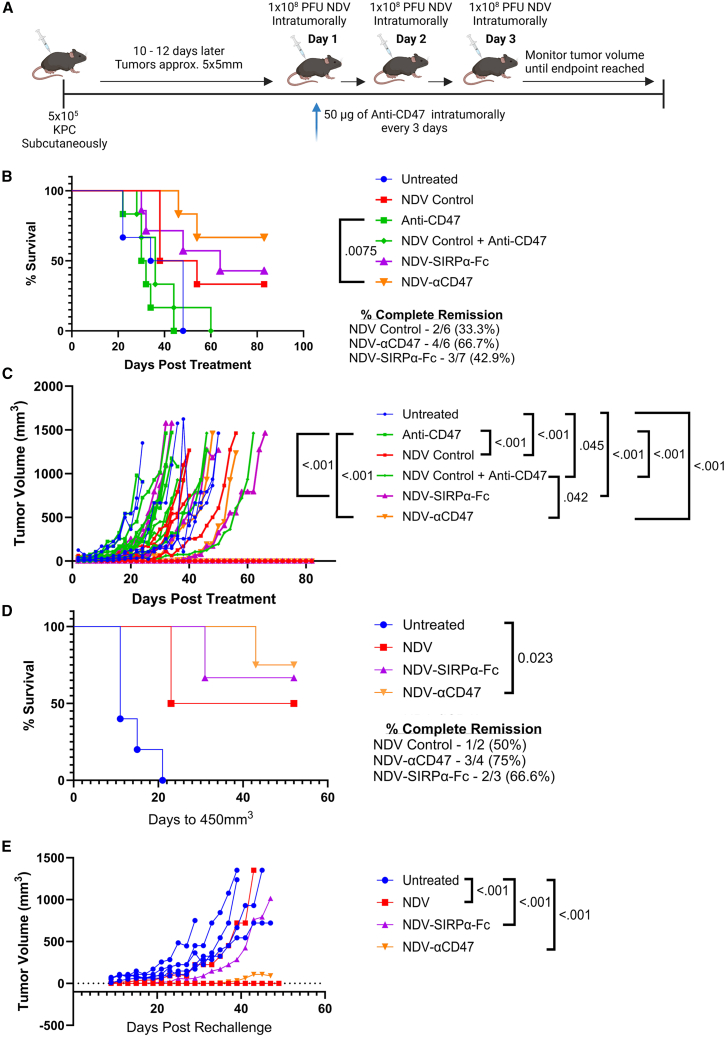
Evaluation of NDV-mediated CD47 blockade in KPC PDAC (A) Schematic outlining of the KPC tumor model. C57BL/6 mice (*n* = 6–8 per group) bearing subcutaneous KPC tumors (∼5 × 5 mm) were left untreated or treated when tumors reached this size. Mice received one of the following regimens: PBS, 1 × 10^8^ PFU NDV-Luciferase, 1 × 10^8^ PFU NDV-αCD47, 1 × 10^8^ PFU NDV-SIRPα-Fc, or 1 × 10^8^ PFU NDV-Luciferase combined with 50 μg anti-CD47 monoclonal antibody (mAb). NDV vectors were administered intratumorally once daily for 3 consecutive days, and anti-CD47 mAb was administered every 3 days for a total of 3 doses. (B) Kaplan-Meier survival curve of KPC-tumor-bearing mice. (C) Tumor volumes measured every other day [(L)×(W)^2^]/2. (D–E) Mice exhibiting complete remission were challenged 63–77 days later in the opposite flank with 5×10^5^ KPC cells (*n* = 2–8). (D) Kaplan-Meier survival curve and (E) tumor volumes [(L)×(W)^2^]/2 measured every other day. Shown are means ± SD. Significance was assessed by log rank test with Bonferroni correction (B, D) and Cox proportional hazards regression (B) or two-way ANOVA with Tukey’s multiple comparison (C, E).

Appreciating that tumor-intrinsic characteristics play a significant role in dictating susceptibility to immunotherapy, we sought to investigate the synergy of NDV and CD47 blockade in a second cancer model of pancreatic ductal adenocarcinoma. KPC cells exhibited the second largest increase in CD47 upregulation and transgene expression following NDV infection (Figure 1A), thus we chose to utilize this model (Figure 6A). Following Kaplan-Meier analysis, a significant survival benefit was only observed in KPC-tumor-bearing mice treated with NDV-αCD47, compared to those treated with anti-CD47 (Figure 6B). No significant survival was observed between NDV alone and NDV-vectored CD47 blockade. Cox proportional hazards regression also indicated NDV and NDV plus CD47 blockade mediated a significant survival benefit when compared to untreated mice or mice treated with anti-CD47 alone (Figure S7). NDV in combination with recombinant anti-CD47 given independently also significantly reduced the hazard of death relative to rNDVs (Figure S7). However, mice receiving NDV-vectorized CD47 blockade had a higher incidence of CR compared to those receiving NDV alone, in addition to delayed tumor growth (Figures 6B and 6C); 66.7% (4/6) and 42.9% (3/7) of mice receiving NDV-αCD47 and NDV-SIRPα-Fc experienced CR, compared to 33.3% (2/6) in those receiving NDV alone (Figure 6B). Mice that experienced CR had undetectable tumors for 63–77 days prior to re-challenge in the opposite flank with 5×10^5^ KPC cells. Compared to age-matched untreated mice (*n* = 5), mice that had previously received NDV and NDV-vectored CD47 blockade exhibited a greater resistance to re-challenge (Figures 6D and 6E). Of those receiving NDV-αCD47 and NDV-SIRPα-Fc, 75% (3/4) and 66.6% (2/3) resisted re-challenge compared to 50% (1/2) of mice that received NDV alone, with NDV-αCD47 treated mice having a significant survival benefit (Figure 6D). Mice that had received NDV and CD47 blockade appeared to have delayed tumor growth as well relative to those that were not treated (Figure 6E). Overall, KPC-tumor-bearing mice that received NDV-vectored CD47 blockade experienced a higher incidence of CR and were more resistant to re-challenge.

## Discussion

Oncolytic viruses induce a pro-inflammatory tumor environment that supports anti-tumor immune responses, and NDV is no exception.^6^^,^^19^^,^^35^^,^^36^ This is highlighted by increased presence of cytokines like interferons (IFNs) and tumor necrosis factor alpha (TNF-α), produced by tumor cells and responding immune cells, which support the activity of immune cells and impair tumor growth.^37^^,^^38^^,^^39^ Production of these cytokines is largely mediated by nuclear factor κB (NF-κB), in response to Toll-like receptors activated by NDV infection.^40^^,^^41^^,^^42^^,^^43^ Upstream of the CD47 reading frame are two super-enhancers, activated by NF-κB to promote CD47 expression.^25^ Therefore, it is likely that the observed increase in CD47 expression following NDV infection of melanoma, pancreatic, and ovarian cancers (Figure 1) was mediated by NF-κB activation. Similar increases were also observed following infection with an oncolytic vaccinia or adenovirus.^44^^,^^45^ There was a direct correlation between the number of infected cells *in vitro* and the level of CD47 expression, suggesting that greater NDV infection resulted in elevated NF-κB activity and higher CD47 expression (Figure 1). The same increases in tumor cell CD47 expression were not apparent following infection *in vivo*; however, increased baseline expression of tumoral CD47 was observed compared to tumor cells grown *in vitro.* This may be attributed to the presence of immune selection pressures *in vivo*, which are absent *in vitro.* Since CD47 expression is linked to inflammation, the CD47/SIRPα signaling axis stands to impact the efficacy of numerous immunotherapies. CD47 expression leads to reduced phagocytosis by APCs during inflammatory conditions, and finding ways to mitigate this is a valuable strategy to support the development of anti-tumor immune responses prompted by cancer immunotherapies, such as oncolytic NDV.

Oncolytic viruses have been successfully engineered to mediate tumor-specific delivery of immune checkpoint blockades (ICBs), helping prevent some of the adverse effects associated with systemic administration of ICB, while improving the anti-tumor immune response initiated by the OV.^6^^,^^19^ Most combined NDV/ICB therapies target signaling pathways that impact T cells, or their interaction with APCs, rather than the interaction of APCs with tumor cells as evaluated here.^6^^,^^19^ Like previously described, NDV-mediated combined immunotherapies, NDV-αCD47 and NDV-SIRPα-Fc, express CD47-blocking agents, with limited impact on viral fitness (Figure S2).^6^^,^^19^^,^^46^ Investigating the blockade of CD47 in the B16-F10 melanoma model showed there was decreased CD47 expression relative to NDV alone presumably due to anti-CD47 and SIRPα-Fc binding of tumoral CD47 and receptor internalization or blockade of SIRPα binding. Additionally, there was no survival benefit afforded when NDV was combined with CD47 blockade (Figure 3B). NDV-SIRPα-Fc did not provide a survival benefit compared to untreated mice, even though it was expressed at greater levels than anti-CD47 (Figures 2 and 3). This could be attributed to differences in binding affinity or in Fc receptor function compared to the anti-CD47 mAb but would require further investigation to confirm. Considering CD47 levels were decreased to background levels with the use of NDV-αCD47, NDV-SIRPα-Fc, and NDV in combination with recombinant anti-CD47 given independently, it is unlikely that this lack of synergy is attributed to the quantity of anti-CD47 or SIRPα-Fc produced or on-target, off-tumor specificity.^47^ This prompted the evaluation of the cells that express the cognate receptor SIRPα, APCs. The mechanism behind the efficacy of CD47 blockade is largely attributed to the improved activity of APCs, which led to improved anti-tumor T cell responses.^24^^,^^28^^,^^48^ APC function can be evaluated by their ability to (1) phagocytose tumor cells, (2) express co-stimulatory markers that support T cell activation, (3) secrete T cell activating cytokines, and (4) present antigen via major histocompatibility complex (MHC) class I and MHC class II to T cells.^49^^,^^50^^,^^51^ As NDV promotes the recruitment of various APC subsets, with peak migration to the tdLN at 24 h post-infection, that time point was chosen to better characterize APC function (Figure S5). APCs extracted from tdLNs have been subjected to the complexity of the tumor and its microenvironment, making their responses to the various treatments more reflective of what would be occurring clinically. Even though isolating APCs from tdLNs and testing them ex vivo (outside the animal but after in vivo exposure) introduces more experimental variation than using purely in vitro–generated cells, this approach yields data that are more representative of what would happen clinically. Four prominent APCs were evaluated: cDC1, cDC2, pDCs, and macrophages. Ultimately, all four cell types did not show significant increases in their recruitment, phagocytosis, or expression of co-stimulatory markers when exposed to NDV and CD47 blockade compared to NDV alone (Figures 4A–4C). Although there was a trend toward increases in the three aforementioned categories when NDV was administered, the only significant increases were observed when assessing macrophage phagocytosis of tumor cells (Figure 4B). APC functionality was further assessed by co-culturing bulk lymphocytes from tdLNs of B16-F10-zsGreen-GP33 mice with bulk splenocytes of P14 mice, which carry a T cell receptor (TCR) specific to the MHC class I immunodominant epitope of GP33.^52^ This appreciates that an APC must have performed all four of the tasks above to effectively activate T cells. T cell activation was assessed by their proliferation and expansion of the T cell effector population (Figures 4D and 4E). These results were in line with those observed in the direct characterization of the APC subtypes (Figures 4A–4C). This contradicts the expected outcomes when CD47 blockade is employed.^24^^,^^53^^,^^54^^,^^55^ Considering that APC function was not improved, this may suggest that therapeutic levels of CD47-blocking agents were not being reached, or there remains other factors limiting APC function.

CD47 blockade targets an innate immune signaling pathway and has been observed to synergize with PD-L1/PD-1 blockade, prompting investigation of this adaptive immune checkpoint. No significant changes relative to NDV alone were observed in PD-L1 expression by APC populations; however, a trend of increased PD-L1 expression in all mice receiving treatment was observed, with macrophages and pDCs exhibiting significantly higher PD-L1 expression (Figure 4G). Effector CD8 T cells from combined NDV and CD47 blockade exhibited greater expression of PD-1 than mice receiving only NDV (Figure 4F). PD-1 is regarded as a marker of T cell activation and T cell exhaustion, increasing as TCR activation occurs and persists.^56^^,^^57^ Given the acute timeline employed, this upregulation likely reflects a more potently activated CD8 T cell population.^56^ This indicates why synergy is often observed with the combination of CD47/SIRPα and PD-L1/PD-1 blockade.^33^ Blocking the interaction of CD47 with SIRPα reduces Src homology region 2 domain-containing phosphatase 1 (SHP-1) and SHP-2 activation, restoring the anti-tumor activities of APCs.^58^^,^^59^ Considering that limited synergy was observed when CD47 and PD-L1 blockades were combined here, additional tumor-intrinsic characteristics, such as production of anti-inflammatory cytokines, loss of antigen expression, or the presence of immunosuppressive cell types, may further limit the efficacy of anti-tumor immune responses.^60^^,^^61^^,^^62^^,^^63^^,^^64^^,^^65^ This is highlighted by the fact that despite the high expression of PD-L1 on B16-F10 tumors, they remain resistant to PD-L1/PD-1 blockade.^66^^,^^67^ Additionally, lymphocytes from the tdLN of untreated tumors also prompted T cell expansion and activation, further suggesting that anti-tumor immune responses are limited through other means.

Considering the differences in tumor characteristics in antagonizing anti-tumor immunity, the combination of NDV and CD47 blockade was also explored in KPC PDAC. Again, no significant survival benefit was extended to mice receiving NDV and CD47 blockade relative to the NDV control. However, mice receiving NDV and NDV-vectored CD47 blockade exhibited CR and were resistant to rechallenge (Figure 5B). In part, this could be attributed to differences in the immune compartment, with KPC PDAC containing high levels of macrophages and myeloid-derived suppressor cells compared to B16-F10, potentially resulting in a tumor microenvironment more sensitive to CD47 immune checkpoint blockade when combined with NDV but not when anti-CD47 is administered alone as it appears to almost negatively impact survival.^68^^,^^69^^,^^70^ However, it should be noted that the small number of animals achieving tumor clearance makes it difficult to make concrete conclusions and is a limitation of our study. Overall, the differences between tumor models in their response to NDV and CD47 blockade highlight the importance of identifying tumor characteristics that predict susceptibility to different immunotherapeutic approaches. Furthermore, tumor responsiveness to combined OV and CD47 blockade immunotherapy has been employed with large, double-stranded DNA viruses, resulting in some success in different tumor types.^44^^,^^45^^,^^71^^,^^72^^,^^73^^,^^74^^,^^75^^,^^76^ Often observed is a delay in tumor volume increases, with a survival benefit afforded in only some cases relative to the vector only control. One group has shown synergy between oncolytic adenovirus and CD47 blockade in B16-F10 melanoma; however, it was later showed that the pH of the tumor microenvironment limits the synergy of this combination.^71^^,^^72^ This supports the idea that the lack of a survival benefit conferred by the combination of NDV and CD47 blockade is more model dependent than vector dependent.

Lastly, it was observed that NDV-vectorized expression of SIRPα-Fc was more efficient than expression of the anti-CD47 mAb. This could potentially be because the anti-CD47 mAb transgene was engineered with the heavy- and light-chain coding sequences separated by an F2A self-cleaving peptide, necessitating efficient co-translational cleavage to generate a functional mAb. Production of a full-length antibody under these conditions likely imposes greater molecular and cellular demands than expression of a single Fc-fusion protein. For example, incomplete ribosomal skipping can lead to uncleaved fusion products, leading to an improper stoichiometry of heavy and light chains and inaccurate inter-chain disulfide pairing, which could increase the burden on the ER folding and quality-control machinery. By contrast, a single-chain Fc-fusion construct is translated as a contiguous polypeptide and does not require a co-translational cleavage event, thereby simplifying folding, assembly, and secretion and reducing dependence on stoichiometric co-expression.

Overall, this study has reinforced the link between OV-mediated inflammation and CD47 expression, highlighting this as an opportunity to enhance innate and downstream adaptive anti-tumor immune responses. While rNDV vectors mediate a functional and localized CD47 blockade that result in improved CD8^+^ T cell activation, this combination did not result in significantly enhanced APC function or survival in B16-F10 melanoma. Further combination with PD-L1 blockade did not synergize but improved the incidence of CR. Similarly, an increased incidence of CR and a resistance to rechallenge were observed when NDV and NDV-vectored CD47 blockade were used in KPC PDAC. Ultimately, these results emphasize context-dependent efficacy of NDV-mediated CD47 blockade and highlight the impact of tumor intrinsic characteristics in determining responsiveness to immunotherapy.

## Materials and methods

### Ethics statement

All mouse experiments were performed in compliance with the guidelines established by the Canadian Council on Animal Care. Animal utilization protocol #4664 was approved by the Animal Care Committee of the University of Guelph. Six-week-old C57BL/6 mice (Charles River Laboratories, MA) were housed in an SPF isolation facility at the University of Guelph under standard conditions: room temperature (22 ± 2°C), hygrometry (55% ± 10%), 12-h light/dark cycle (light 7 am to 7 pm), air replacement (15–20 volumes/h), and access to water and food ad libitum. These mice were allowed to acclimate for at least 7 days before experimentation. Isoflurane anesthesia was used during administration of all cells and vectors, and all efforts to minimize suffering were taken.

### Cells

Cells obtained from ATCC (VA) include chicken fibroblast DF-1 (CRL-12203), murine melanoma B16-F10 (CRL-6475), HeLa derived HEp-2 (CCL-23), African green monkey kidney Vero cells (CCL-81), and human embryonic kidney 293 (CRL-1573) and 293T cells (CRL-3126). The remaining cells were generously donated: murine ovarian cancer ID8 and murine pancreatic KPC (Dr. Jim Petrik, University of Guelph, Canada), murine breast cancer 4T1 (Dr. Samuel T. Workenhe, University of Guelph, Canada), and human melanomas SKMEL-28 and UACC-62 (Dr. John Bell, Ottawa Hospital Research Institute, Canada). All cells were cultured in Dulbecco’s modified Eagle medium (DMEM) (Cytiva, MA), except 4T1 and KPC, which were cultured in Roswell Park Memorial Institute (RPMI) medium (Cytiva, MA). Culture media was supplemented with 5% fetal bovine serum (FBS) (Gibco, MA) and 2mM L-Glutamine (Thermo Fisher Scientific, MA) aside from DF-1 media containing 10% FBS and cultured at 37°C and 10% CO_2_.

### Newcastle disease viruses

Mesogenic NDV-GFP, NDV-Luciferase, NDV-αCD47, and NDV-SIRPα-Fc bearing a L289A mutation in the F protein were propagated and purified as previously described.^17^^,^^77^ The luciferase or GFP reporter gene and the required NDV-specific RNA transcription signals were inserted between the P and M genes. The cDNA for anti-CD47 comprised of variable heavy- and light-chain sequences from the rat anti-mouse CD47 monoclonal antibody (clone MIAP301) fused to the human IgG1 Fc domain, with heavy and light chains separated by a self-cleaving furin-F2A peptide. The cDNA for SIRPα-Fc was derived from the non-obese diabetic murine SIRPα (aa 6–119) and fused to the CH2-CH3 domain of human IgG1. Both transgenes were human-mouse codon optimized and synthesized by GenScript (NJ). NDV encoding the soluble extracellular domain of PD-1 (aa 21–268, GenBank: X67914.1) has been described previously.^6^ InFusion (Takara Bio, CA) cloning was used to insert these transgenes, flanked by gene initiation and gene termination signals, between the P and M genes of NDV to create NDV-αCD47 and NDV-SIRPα-Fc. rNDVs were rescued as previously described.^77^ Anti-CD47 and SIRPα-Fc were also cloned into expression plasmids containing a ubiquitous CASI promoter.^78^ NDV vectors were titered by TCID_50_, using the Spearman-Kärber titer calculator to generate titers in PFU/mL.^17^ Vero cells were seeded at 2 × 10^4^ cells per well in 80 μL of infectious media (2% FBS, 125 μg/mL Trypsin (Cytiva, MA), 100 U/mL Penicillin, and 100 μg/mL Streptomycin (Cytiva, MA) and infected with 20 μL of 10-fold serial dilutions of viral stocks. Immunofluorescence assay was performed 3–5 days later, as previously described.^17^

### Lentivirus

Murine CD47 cDNA was generated following RNA isolation from C57BL/6 splenocytes using GenElute Viral RNA Miniprep Kit (Sigma-Aldrich, MO), following the manufacturer’s protocol. Murine CD47 (Forward: 5′-aattgatccttcgaactagtCC**GCCACC**ATGTGGCCCTTGGCGGCGGCGC-3′, Reverse: 5′-CCTATTCCTAGGAGGTTGGATAGTCC-3′) and mCherry (Forward: 5′-TCCAACCTCCTAGGAATAGGcgaaaaagaagatcaggttcgggtgcg-3′, Reverse: 5′-gccctagatgcatgcggatcCTACTTGTACAGCTCGTCCATGCCGC-3′) separated by a self-cleaving furin-F2A peptide were cloned into a pSIN lentiviral vector encoding puromycin resistance using infusion cloning. pSIN-CD47-mCherry, psPAX2 helper plasmid, and pCI-VSVG were transfected into HEK293T cells at a 1:1:1 ratio using Polyethylenimine max (Kyfora Bio LLC, PA) and Opti-mem (Gibco, MA). Supernatant was collected 48 h post-transfection and 0.45 μM filtered before storage at −80°C. HEK 293T cells were transduced with CD47-mCherry lentivirus for 24 h before selection with 1 μg/mL Puromycin (Gibco, MA).

### *In vitro* infection of tumor cells for flow cytometric analysis

ID8, B16-F10, KPC, SKMEL-28, or UACC-62 cells were lifted using 10mM EDTA-PBS prior to infection, in triplicate, at MOIs of 1 and 0.1 with NDV-GFP in round-bottom 96-well plates (650180, Greiner Bio-One, Austria) (2 × 10^4^ cells/well). After 6, 12, 24, or 48 h, cells were detached using 10mM EDTA-PBS and washed in 0.5% Bovine Serum Albumin (Thermo Fisher Scientific, MA) (BSA)-PBS (fluorescence-activated cell sorting [FACS]). Cells were stained with a combination of 7-AAD, Anti-CD16/CD32, PE-Anti-CD47, SparkRed718 anti-PD-L1, or AlexaFluor647-Anti-Calreticulin as indicated in Table S7 (BioLegend, CA). Cells were collected on a BD FACS Canto II (BD, NJ) or a Cytek Northern Lights (Cytek, CA) flow cytometer and analyzed using FlowJo v.10.1.

### Quantifying anti-CD47 and SIRPα-Fc transgene expression

Biological replicates of B16-F10 (*n* = 5), UACC-62 (*n* = 3), or SKMEL-28 (*n* = 4) cells were infected at an MOI of 1 in 6-well plates (1 × 10^6^ cells/well) for 48 h after which supernatants were collected and stored at −80°C. Concentration of anti-CD47 and SIRPα-Fc was determined by human IgG ELISA (Abcam, USA) using the manufacturers protocol.

### Western blot analysis of anti-CD47 and SIRPα-Fc transgene expression

Equal volumes of allantoic fluid from SPF embryonated chicken eggs infected with NDV-GFP, NDV-αCD47, or NDV-SIRPα-Fc were mixed with 6× loading dye containing 30 μL β-mercaptoethanol, heated at 95°C for 10 min, cooled on ice, and then resolved on a 4%–16% SDS-PAGE gradient gel. Following transfer onto PVDF, membranes were blocked overnight at 4°C with 5% skim milk, 0.1% PBS-Tween 20 (PBS-T), then washed with 0.1% PBS-T and incubated with primary goat anti-human-IgG (1/2,000) (31125, Invitrogen, USA) for 1 h at room temperature. Blots were washed again with 0.1% PBS-T before incubation with rabbit anti-goat horseradish peroxidase (HRP)-conjugated secondary antibody (1/5,000) (81–1620, Invitrogen, USA) for 1 h at room temperature. Protein was detected by Pierce SuperSignal West Pico PLUS Chemiluminescent Substrate (Thermo Fisher Scientific, MA) and the BioRad ChemiDoc MP Imaging System, Image Lab 6.1 Software.

### Multi-step growth curves

B16-F10, UACC-62, and SKMEL-28 cells were infected in triplicate at an MOI of 0.1 in 12-well plates (5 × 10^5^ cells/well) and supernatant collected at 24, 48, and 72 h post-infection. Viral titer was determined by TCID_50_ as described above.

### Metabolic activity assay

B16-F10, UACC-62, and SKMEL-28 cells were infected in triplicate at MOIs of 1, 10, and 100 for 24, 48, or 72 h in 96-well plates (5 × 10^3^ cells/well). Resazurin (resazurin sodium salt; Sigma-Aldrich, MO) was added to a final concentration of 0.025 μg/μL for 2 h prior to quantification of resorufin using a PerkinElmer Enspire Multimode plate reader spectrophotometer (PerkinElmer, CT) with an excitation/emission of 530/590 nm.

### CD47 binding assay

Anti-CD47 and SIRPα-Fc expression plasmids were transfected into HEK293 cells using PEI Max. Twenty-four hours post-transfection, media was exchanged and replaced with basal DMEM to remove FBS. Four days later, supernatant was collected, and antibodies were purified using HiTrap ProteinG columns (MilliporeSigma, MA) following the manufacturer’s protocol. Antibody concentration was determined using an anti-human IgG ELISA (Abcam, UK) following the manufacturer’s protocol. HEK293T-CD47-mCh cells were collected by addition of 10 mM EDTA-PBS. Cells were washed in FACS buffer before incubation, in triplicate, with varying micrograms of purified anti-CD47 or SIRPα-Fc for 30 min at 4°C. Cells were then stained with anti-CD16/32, 7-AAD, PE-Anti-CD47, and PE-Cy7-Anti-Human IgG (1/200) (BioLegend, CA) (Table S7), processed on a Cytek Northern Lights flow cytometer, and analyzed using FlowJo v.10.1.

### Functional binding assay

Half well plates were coated overnight at 4°C with 100 ng of CD47 (ab231160, Abcam, UK). Wells were washed three times with 0.2% PBS-Tween 20 before incubation in Superblock blocking buffer (37515, Thermo Fisher Scientific, MA) for 1 h at room temperature. Thirty microliters of 2-fold serially diluted allantoic fluid, ranging from undiluted to 1/2,048, from NDV-αCD47-, NDV-SIRPα-Fc-, or NDV-GFP-infected SPF embryonated chicken eggs was added to each well for 1 h at 37°C (*n* = 3). Wells were washed three times with 0.1% PBS-Tween 20 before addition of goat anti-human IgG HRP conjugated secondary antibody diluted 1/2,500 (31410, Thermo Fisher Scientific, MA) for 1 h at 37°C. Cells were washed three times with 0.2% PBS-Tween 20, then TMB substrate (34021, Thermo Fisher Scientific, MA) was added, and emission at 600 nm was measured using a PerkinElmer Enspire Multimode plate reader spectrophotometer.

### Tumor models

For the murine melanoma model, 2 × 10^5^ B16-F10, B16-F10-GFP, or B16-F10-zsGreen-GP33 cells suspended in 20 μL of PBS were implanted intradermally into 6-week-old C57BL/6 female mice in 20 μL. Use of B16-F10-GFP and B16-F10-zsGreen-GP33 allow for the identification of tumor cells in the generation of single-cell suspensions from tumor cells as per GFP positivity and for the detection of phagocytosed tumor cells. Use of GP33 enables tracking of antigen presentation to T cells by APCs. For the pancreatic ductal adenocarcinoma model, 5 × 10^5^ KPC cells were injected subcutaneously into 6-week-old C57BL/6 mice in a volume of 100 μL. For both models, when tumors were approximately 5 × 5 mm, mice were either PBS treated or received one of the following: three consecutive intratumoral doses of 1 × 10^8^ PFU NDV-Luciferase, NDV-αCD47, NDV-SIRPα-Fc, NDV-Luc plus 50 μg anti-CD47 (BE0270, BioXCell, CT), 50 μg anti-CD47 only, or 50 μg of a rat isotype control (BE0089, BioXCell, CT) (*n* = 6–8 per treatment group). Mice receiving anti-CD47 antibody did so every 3 days for the duration of the study. For the combined CD47 and PD-L1 blockade study, 2 × 10^8^ PFU total NDV was administered, consisting of the following: 2 × 10^8^ PFU NDV-Luciferase, 1 × 10^8^ PFU NDV-αCD47 plus 1×10^8^ NDV-Luciferase, 1 × 10^8^ NDV-sPD1 plus 1 × 10^8^ NDV-Luciferase, or 1 × 10^8^ PFU NDV-αCD47 plus 1 × 10^8^ PFU NDV-sPD1. Tumors were measured every other day and tumor volume determined using the equation [(L)×(W)^2^]/2. Mice were euthanized once tumors reached 15 mm in any one direction or at the indicated time point. Significance was assessed using Kaplan-Meier and Cox Proportional hazards.

### Tissue processing

Single-cell suspensions were generated from tumors using GentleMACs C tubes (130-093-237, Miltenyi Biotec, Germany) following the manufacturer’s protocol for isolation of immune cells or isolation of immune and tumor cells (Miltenyi Biotec, Germany). Single-cell suspensions from spleen or tumor-draining inguinal lymph node were generated by placing the lymph node or spleen in a 6-well dish and pressing it with the back of a 3 mL plunger (Thermo Fisher Scientific, MA). Plunger and well were rinsed with Hanks’ Balance Sodium Salt (HBSS) (Cytiva, MA), and the cell suspension was passed through a 100 μm filter (Thermo Fisher Scientific, MA) before centrifugation at 500 × g for 5 min and resuspension in RPMI containing 10% FBS, 100 U Penicillin, 100 μg/mL Streptomycin, and 2 mM L-Glutamine. ACK Lysis Buffer (150 mM NH_4_Cl, 10 mM KHCO_3_, 0.1 mM Na_2_EDTA, pH 7.3) was used to lyse red blood cells as needed. Briefly, cells were pelleted at 500 × g for 5 min and resuspended in 2 mL of ACK lysis buffer for 5 min before addition of 2 mL HBSS. Cells were pelleted at 500 × g for 5 min and resuspended in RPMI as described above. All single-cell suspensions were diluted accordingly and mixed 1:1 with 0.4% Trypan blue (Gibco, MA) to determine cell viability prior to further experimentation.

### Surface staining of single-cell suspensions

Single-cell suspensions, from tumors or tumor-draining inguinal lymph nodes, were pelleted at 500 × g for 5 min and resuspended in FACS buffer. Cells were pelleted again and resuspended in FACS buffer containing anti-CD16/32 (BioLegend, CA) for 20 min at 4°C. Cells were then pelleted and resuspended in FACS containing a combination of the antibodies listed in Table S7. Cells were incubated in antibody for 25 min at 4°C before washing in FACS buffer. If viability staining was performed using ZombieNIR (100 μL per sample) (423105, BioLegend, CA), cells were first resuspended in PBS prior to centrifugation at 500 × g for 5 min and staining in ZombieNIR for 25 min at 4°C. After incubation, cells were washed with PBS before centrifugation at 500 × g for 5 min and resuspension in FACS buffer. Cells that were not stained with ZombieNIR were resuspended in FACS immediately. Both were acquired using a BD FACS Canto II or Cytek Northern Light Flow Cytometer, then analyzed using FlowJo v.10.1.

### Splenocyte and lymphocyte co-cultures

Single-cell suspensions were generated from the tumor-draining inguinal lymph node of B16-F10-tumor-bearing mice treated with one of the CD47 blockade therapies described above or from the spleens of naive P14 mice (LCMV p33-specific TCR transgenic P14 mice strain B6,D2-TgN (TcrLCMV0327Sdz, Jackson Laboratories, ME). Bulk P14 splenocytes were dyed with CellTraceViolet (C34557, Thermo Fisher Scientific, MA) as per the manufacturer’s protocol. Briefly, splenocytes were resuspended in 1 mL PBS and stained with 5 μM of CellTraceViolet for 20 min at 37°C in the dark before addition of 5 mL RPMI containing 10% FBS. Cells were pelleted by centrifugation at 500 × g for 5 min before resuspension in RPMI containing 10% FBS, 100 U Penicillin, 100 μg/mL Streptomycin, and 2 mM L-Glutamine. Bulk lymphocytes were co-cultured with bulk splenocytes at a ratio of 1:3 for 24 or 96 h to assess the activation of P14 splenocytes following CD47 blockade. After 24 or 96 h, cells were collected and pelleted by centrifugation at 500 × g for 5 min and resuspended in FACS buffer. Cells were then subjected to the surface staining protocol as described above.

### Flow cytometric analyses

All gating strategies began by FSC-A vs. SSC-A to identify general cells. Doublets were removed by FSC-A vs. FSC-H. Dead cells were excluded by taking 7-AAD or ZombieNIR negative cells, informed by use of unstained controls.

### Identification of antigen-presenting cell subsets

APCs were identified from live cells, as described above, using the following gating strategy. All plots utilize FSC-A on the *x* axis unless indicated otherwise. F4/80^+^ were classified as macrophages. Among the F4/80^–^ population, cells were analyzed for CD11c and B220 expression, with CD11c^+^ B220^+^ cells identified as plasmacytoid dendritic cells. CD11c^+^ cells were further analyzed for MHCII expression, with CD11c^+^ MHCII^+^ cells defined as classical dendritic cells. CD11c vs. XCR1 and CD11c vs. SIRPα were used to identify type I (XCR1^+^) and type II (SIRPα^+^) classical dendritic cells, respectively. Following identification of specific APC lineages, phagocytosis of tumor cells was assessed by GFP positivity and activation by presence of CD80^+^ CD86^+^ double-positive cells or PD-L1 expression as PD-L1^+^.

### Identification of T cells following *ex vivo* co-culture

T cells were identified from live cells, as described above, by the following gating strategy. NK1.1^−^ followed by CD3^+^, then separated into CD4^+^ and CD8^+^ by CD4 vs. CD8. CD8 T cells were assessed for presence of CellTraceViolet (CellTraceViolet^+^) or for expression of CD44 and CD62L. CD44^+^ CD62L^−^ were identified as educated CD8 T cells. PD-1 was then assessed as an activation marker.

### Separating tumor cells from lymphocytes

Tumor cells were identified from live cells as described above, as being GFP+, then CD45−. Tumor cells were further assessed for calreticulin expression. CD45^−^ GFP^+^ cells were then assessed for SIRPα.

### Binding assay

Live cells, identified as described above, were evaluated for mCherry. hIgG-positive cells were then identified from mCherry^+^ cells.

### *In vitro* CD47 expression

Following identification of live cells, CD47^+^, GFP^+^ or calreticulin^+^ cells were identified. As indicated in the figure legend, GFP^+^ or CD47^+^ cells were further assessed from CD47^+^ or GFP^+^ populations.

### Statistical analysis

All results were analyzed and graphed using Prism version 9 (GraphPa, MA). Where appropriate, statistical tests used to determine significance included unpaired parametric *t* tests, two-way ANOVA with Tukey’s multiple comparison, Kaplan-Meier with Bonferroni correction, or Cox proportional hazards regression, as described in the figure legends.

## Data and code availability

All data produced and analyzed in this study are fully available and included in the published article and its supplementary files.

## Acknowledgments

Funding was provided by Natural Sciences and Engineering Research Council (10.13039/501100000038NSERC) of Canada (RGPIN-2018-04737) and the 10.13039/100009326Cancer Research Society (#946085). Stipend support was provided by the 10.13039/100014116Ontario Veterinary College (OVC) (JGEY, LC, ESBC, ANG), the Ontario Graduate Scholarship (OGS) program (JGEY, ANG), Pathobiology Graduate Growth funds (AEB), and the NSERC Postgraduate Scholarship-Doctoral program (JGEY). We thank the technical staff at the University of Guelph animal facility for their animal care services. The graphical abstract was created in BioRender (https://BioRender.com/aqd8fbx).

## Author contributions

Conceptualization, J.G.E.Y, T.M.McA., and S.K.W.; methodology, J.G.E.Y., L.C., E.S.B.C, and S.K.W.; investigation, J.G.E.Y., L.C., A.E.B., E.S.B.C., A.N.G., and M.E.H.; visualization, J.G.E.Y.; formal analysis, J.G.E.Y. and S.K.W.; resources, S.T.W and S.K.W.; writing—original draft preparation, J.G.E.Y.; writing—review and editing, J.G.E.Y., L.C., K.K., S.T.W., and S.K.W.; supervision, L.S., K.K., S.T.W., and S.K.W.; project administration, S.K.W.; funding acquisition, S.K.W. All authors have read and agreed to the published version of the manuscript.

## Declaration of interests

S.K.W. is a scientific co-founder of Avamab Pharma Inc., a pre-clinical, pre-revenue stage company dedicated to research and development of AAV gene therapies for the treatment and prevention of infectious diseases and Inspire Biotherapeutics, a pre-clinical, pre-revenue stage company dedicated to research and development of AAV gene therapies for the treatment of monogenic lung diseases. S.K.W. and L.S. are co-inventors on a pending US and Canadian patent for the engineered Newcastle disease virus vector and uses thereof, filed/owned by the University of Guelph. The funders had no role in the design of the study; collection, analyses, or interpretation of data; writing of the manuscript; or decision to publish the results.

## Supplemental information

Document S1. Figures S1–S7 and Tables S1–S7

Document S2. Article plus supplemental information
