# Supplementary material for: Oncolytic Newcastle-disease-virus-mediated CD47 blockade in preclinical melanoma and pancreatic cancer models
Source: Mol Ther Oncol. 2025 Nov 1;33(4):201076. doi: 10.1016/j.omton.2025.201076 (PMC12657332; doi:10.1016/j.omton.2025.201076)
Supplement: Document S1. Figures S1–S7 and Tables S1–S7 [file mmc1.pdf]

OMTON, Volume *33*

Supplemental information

Oncolytic Newcastle-disease-virus-mediated CD47 blockade in preclinical melanoma and pancreatic cancer models

Jacob G.E. Yates, Lily Chan, Alyssa E. Bogle, Elena S.B. Campbell, Arielle N. Gillies, Madison E. Hughes, Thomas M. McAusland, Leonardo Susta, Khalil Karimi, Samuel T. Workenhe, and Sarah K. Wootton

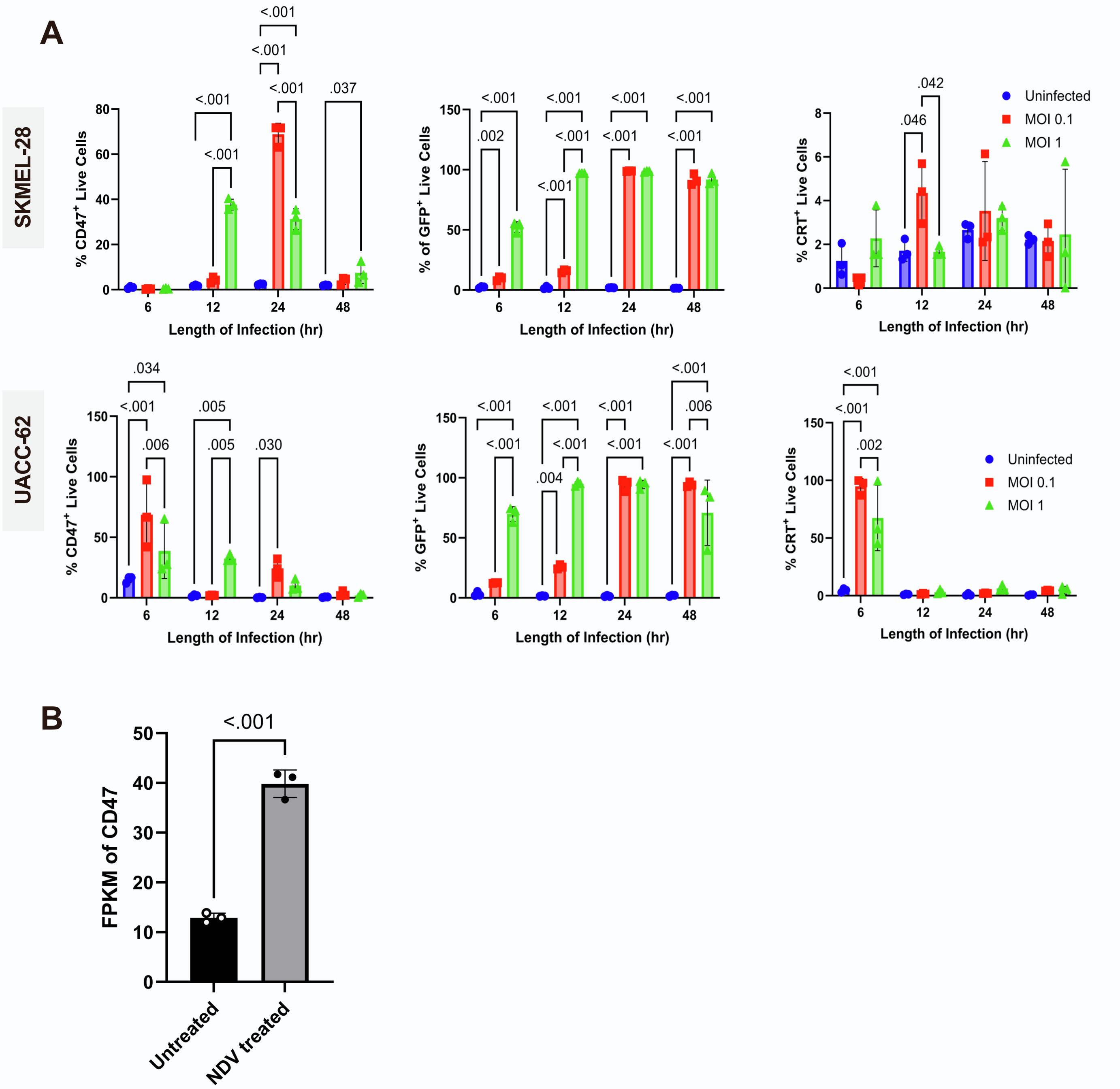

Figure S1. The impact of NDV infection on CD47 expression in human tumors. (A) Human melanoma cell lines SKMEL-28 and UACC-62 were left uninfected or infected at multiplicity of infections of 1 or 0.1 with mesogenic NDV-GFP and harvested 6-, 12-, 24- and 48-hours post infection to assess CD47 expression, viral gene expression and surface expression of calreticulin by flow cytometry. (B) Fragments per kilobase million (FPKM) following RNA sequencing of human glioblastoma (LN229) cells infected with lentogenic NDV (accession PRJNA947071; GEO: GSE227791). Significance was assessed by two-way ANOVA with Tukey's multiple comparisons test (A) and unpaired parametric T-test in (B). Shown are means +/- SD.

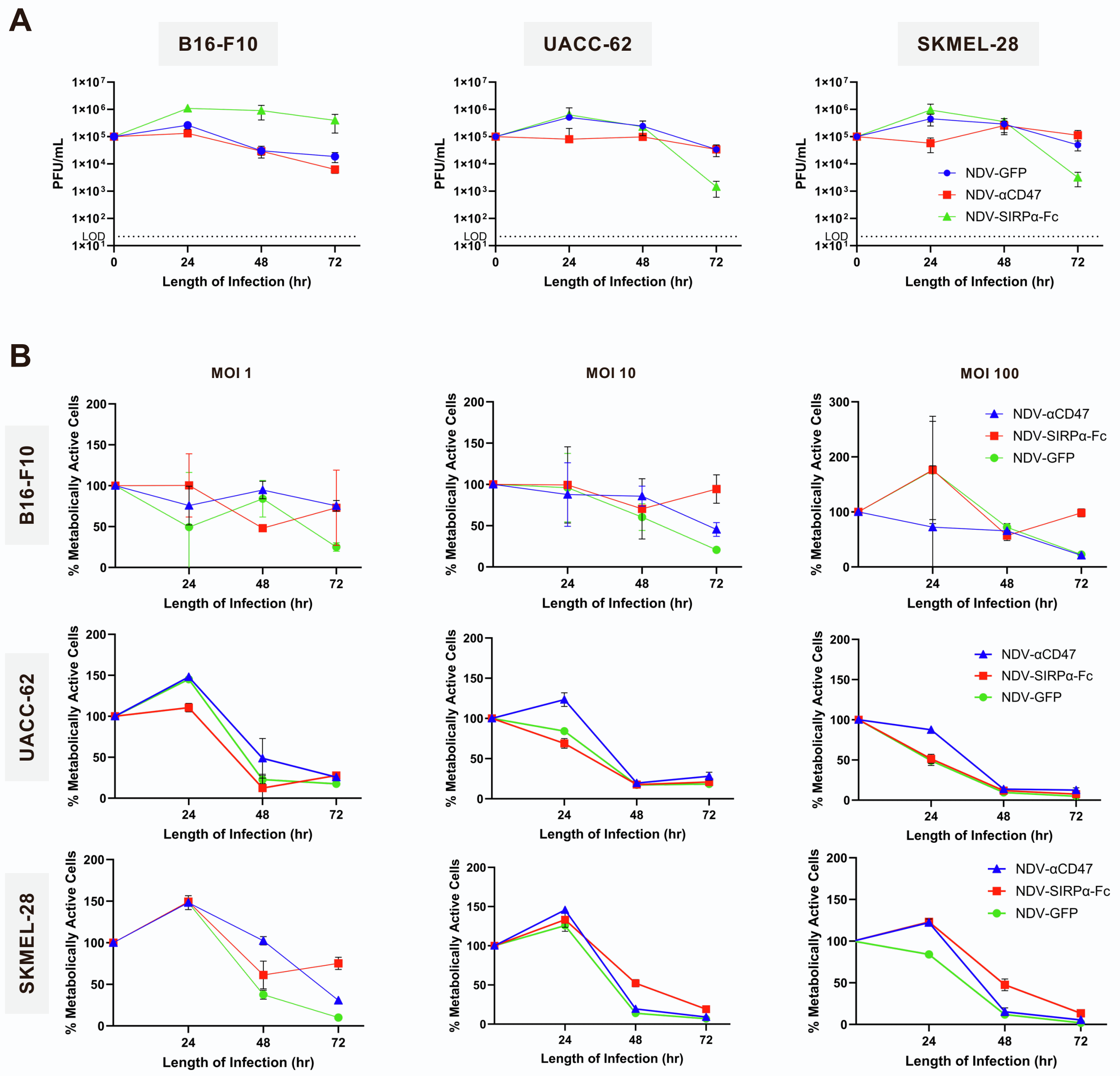

Figure S2. Characterizing rNDV viral fitness. (A) B16-F10, UACC-62 or SKMEL-28 cells were infected, in triplicate, with NDV-GFP, NDV-αCD47 or NDV-SIRPα-Fc at a multiplicity of infection of 0.1 based on the number of cells seeded. A sample of the supernatant was harvested every 24 hours, for a total of 72 hours and virus concentration was determined by $TCID_{50}$ on Vero cells supplemented with 2% FBS and 125 μg/mL Trypsin using the Spearmann-Karber titer calculator. (B) The percent of metabolically active B16-F10, UACC-62 or SKMEL-28 cells following infection with NDV-GFP, NDV-αCD47 or NDV-SIRPα-Fc at multiplicities of infection of 100, 10 and 1 relative to that of uninfected cells, in triplicate, at 24-, 48- and 72-hours post infection. Significance was assessed by two-way ANOVA with Tukey's multiple comparison in and is shown in Supplementary Tables 1 - 3 for (A) and 4 - 6 for (B) Shown are means +/- SD.

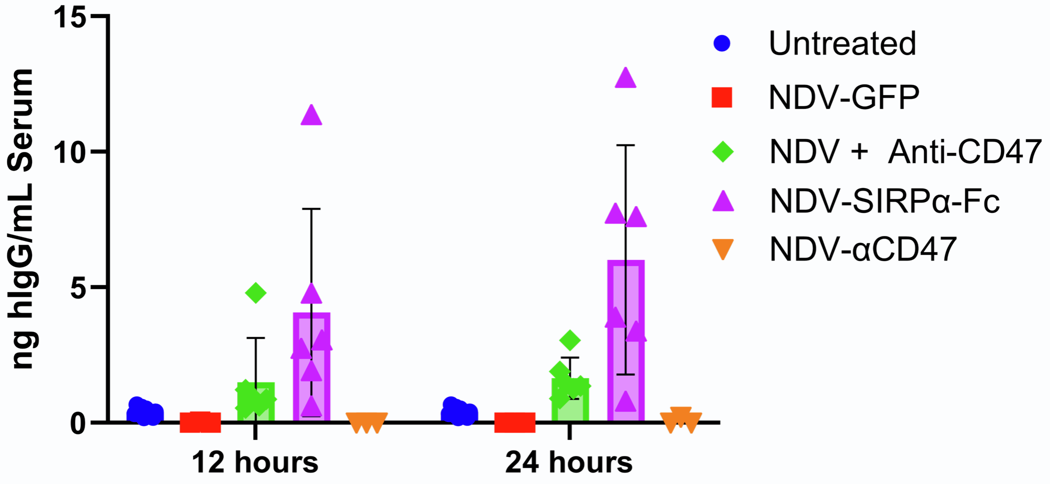

Figure S3. Concentration of anti-CD47 and SIRPα-Fc in serum of B16-F10 tumor bearing mice following treatment with rNDVs. Serum was collected by retro-orbital vein and tissue lysates by homogenization with a ceramic bead in 1.5 mL tubes containing 300 μL of RIPA buffer. Significance was assessed by two-way ANOVA with Tukey's multiple comparison Shown is means +/- SD.

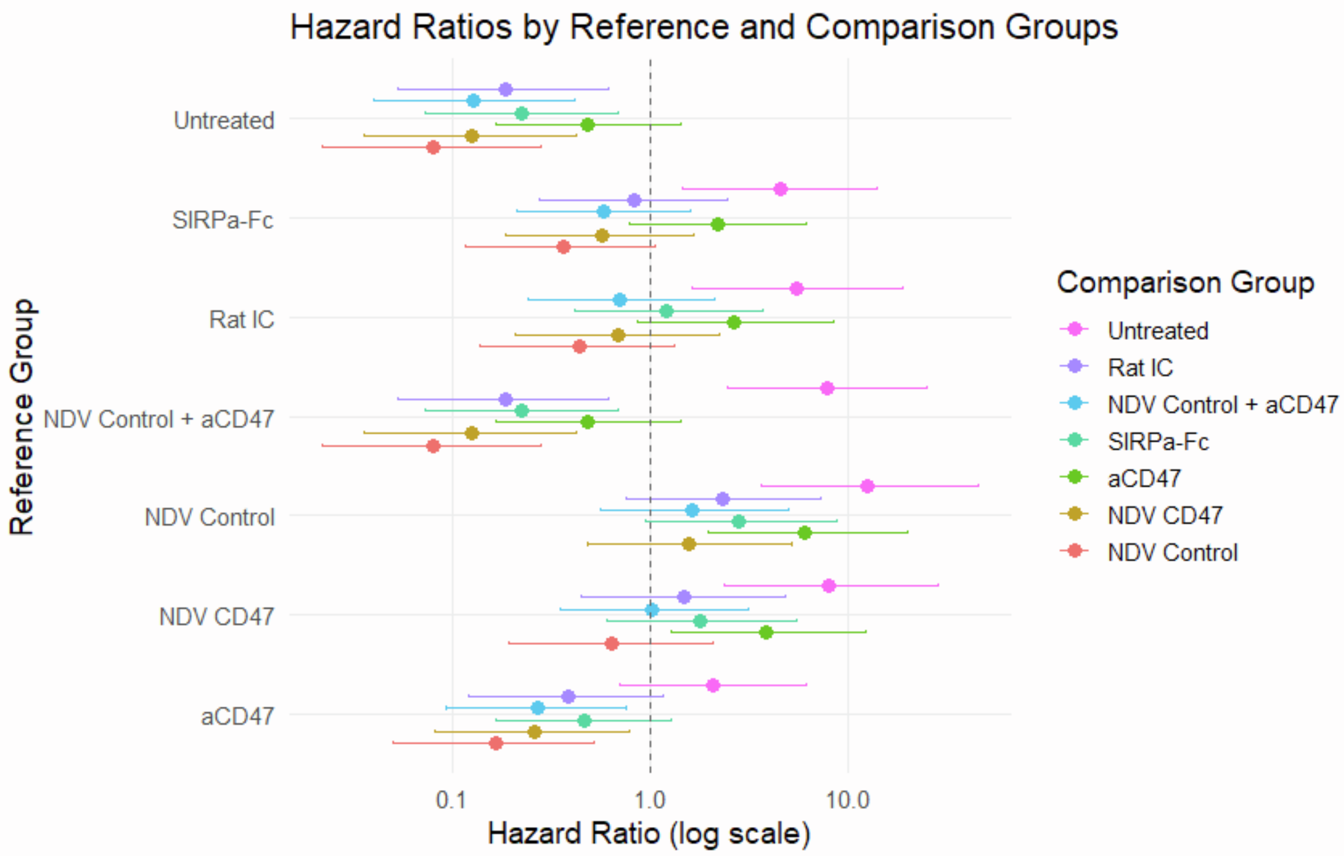

Figure S4. Cox Proportional Hazards of B16-F10 tumor bearing mice following treatment with rNDVs. Hazard ratios (HR) and 95% confidence intervals from univariate Cox proportional hazards regression comparing each treatment group. HR > 1 indicates increased risk of death; HR < 1 indicates improved survival. Analysis was performed using GraphPad Prism.

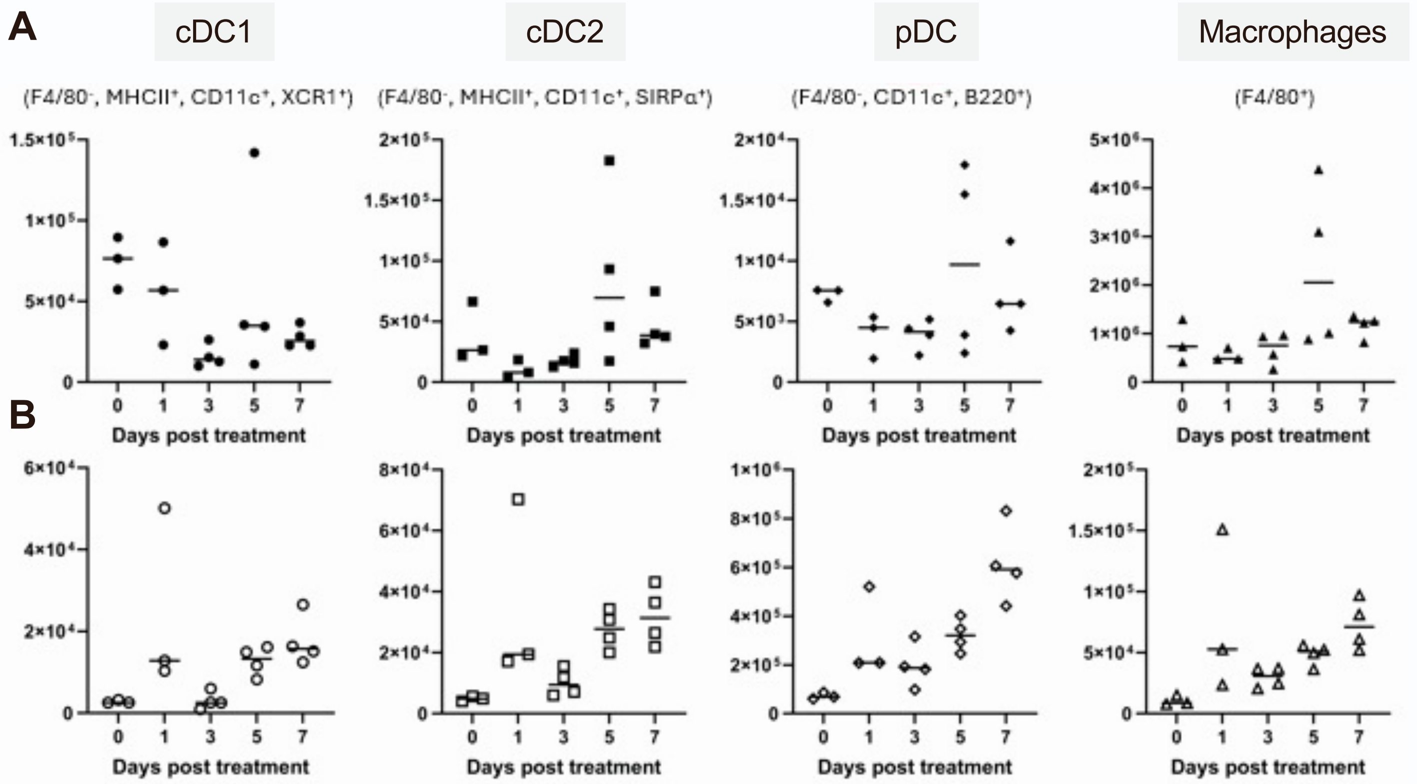

Figure S5. (A) B16-F10-GFP tumor bearing mice were euthanized 0, 1-, 3-, 5- or 7-days post treatment with 1x10[8] PFU NDV-Luciferase and both the tumor and tumor draining lymph node were processed into single cell suspensions using GentleMACs C-tubes and the manufacturers protocol, while lymph nodes were pressed in a 6-well dish with the back of a 3 mL plunger before being passed through a 70 µm filter. Resulting cells were prepared for flow cytometric analysis on a BD Canto II by staining with 7-AAD, APC-Cy7-Anti-MHCII, BrilliantViolet510-Anti-B220, APC-Anti-SIRPα, BrillirantViolet421-Anti-XCR1, PE-Anti-F4/80 and PE-Cy7-Anti-CD11c and analyzed with FlowJo V10.1.

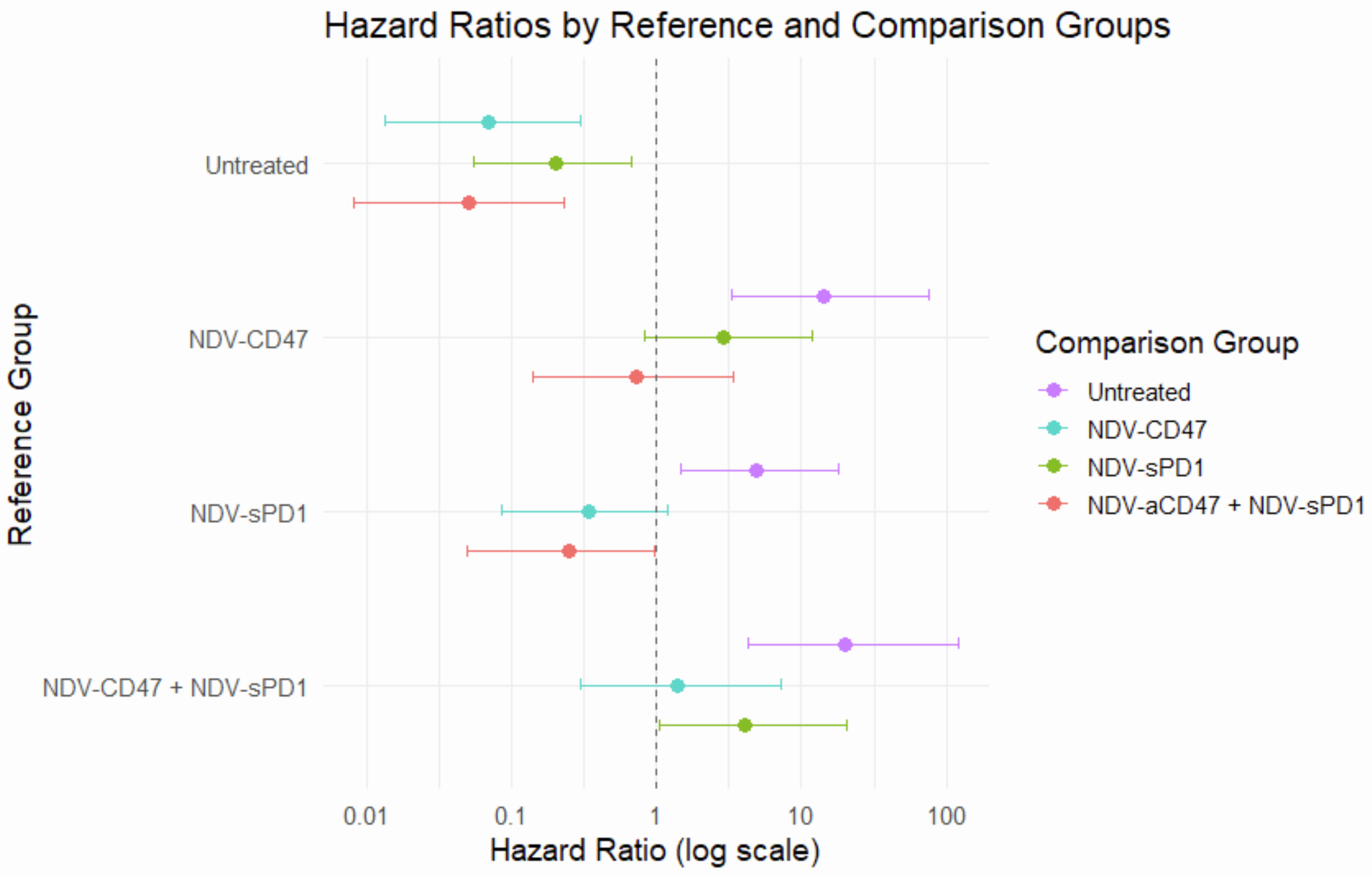

Figure S6. Cox Proportional Hazards of B16-F10 tumor bearing mice following combined anti-CD47 and anti-PD-L1 treatment. Hazard ratios (HR) and 95% confidence intervals from univariate Cox proportional hazards regression comparing each treatment group. HR > 1 indicates increased risk of death; HR < 1 indicates improved survival. Analysis was performed using GraphPad Prism.

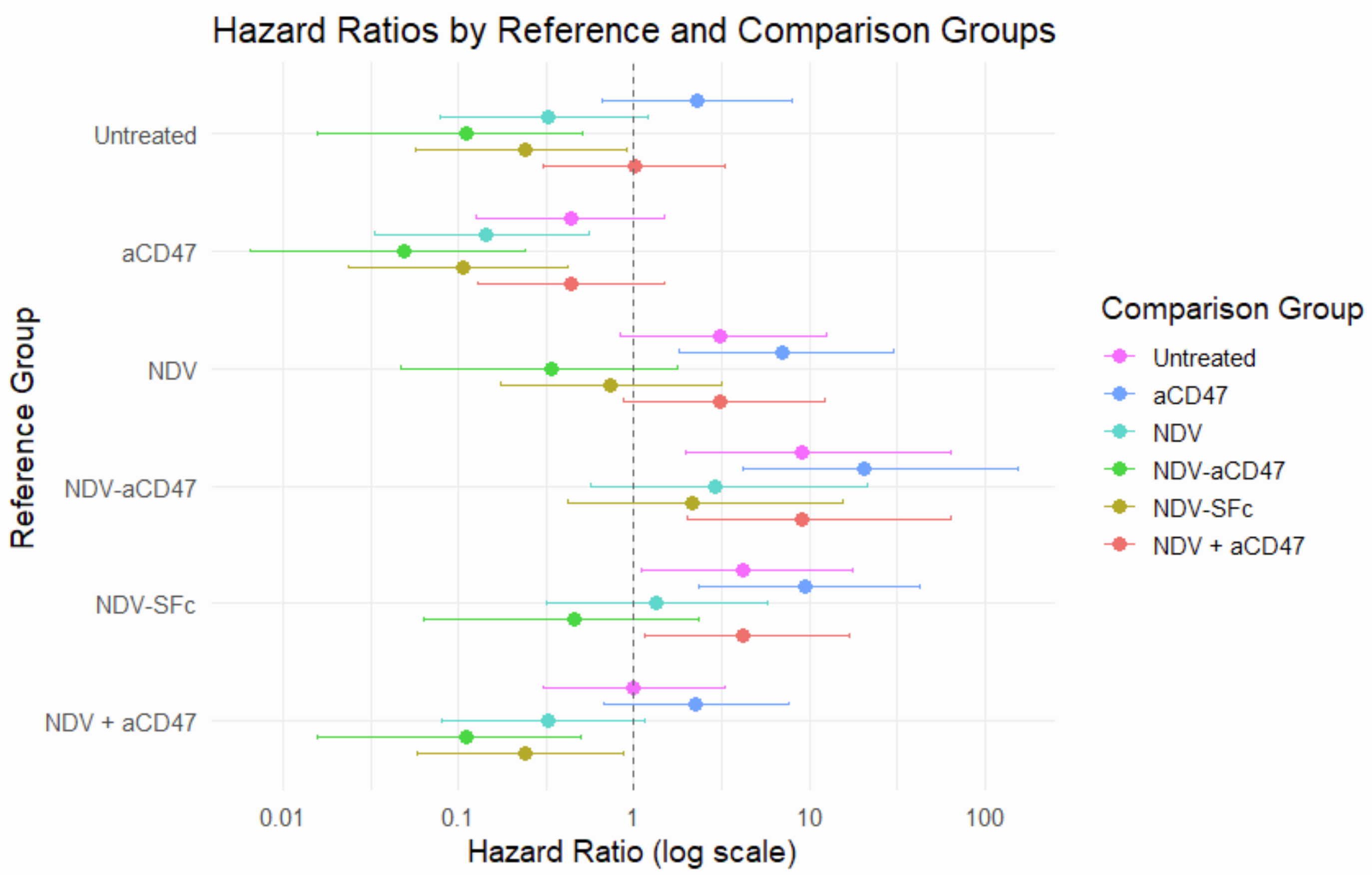

Figure S7. Cox Proportional Hazards of KPC tumor bearing mice following rNDV treatment. Hazard ratios (HR) and 95% confidence intervals from univariate Cox proportional hazards regression comparing each treatment group. HR > 1 indicates increased risk of death; HR < 1 indicates improved survival. Analysis was performed using GraphPad Prism.

Table S1. Significance values associated with multi-step growth curve in B16-F10 cells in Figure S2A. B16-F10 cells were infected, in triplicate, with NDV-GFP, NDV-αCD47 or NDV-SIRPα-Fc at a multiplicity of infection of 0.1 based on the number of cells seeded. A sample of the supernatant was harvested every 24 hours, for a total of 72 hours and virus concentration was determined by $TCID_{50}$ on Vero cells supplemented with 2% FBS and 125 µg/mL Trypsin using the Spearmann-Karber titer calculator. Significance was assessed by two-way ANOVA with Tukey's multiple comparison. * - < 0.05, ** - < 0.01, *** - < 0.001 and **** - < 0.0001.

**B16-F10**	**NDV-SIRPα-Fc 24 hour**	**NDV-SIRPα-Fc 48 hour**	**NDV-SIRPα-Fc 72 hour**
NDV-αCD47 24 hour	< 0.001	N/A	N/A
NDV-GFP 24 hour	< 0.001	N/A	N/A
NDV-αCD47 48 hour	N/A	< 0.001	N/A
NDV-GFP 48 hour	N/A	< 0.001	N/A
NDV-αCD47 72 hour	N/A	N/A	0.021
NDV-GFP 72 hour	N/A	N/A	0.026

Table S2. Significance values associated with multi-step growth curve in UACC-62 cells in Figure S2A. UACC-62 cells were infected, in triplicate, with NDV-GFP, NDV-αCD47 or NDV-SIRPα-Fc at a multiplicity of infection of 0.1 based on the number of cells seeded. A sample of the supernatant was harvested every 24 hours, for a total of 72 hours and virus concentration was determined by $TCID_{50}$ on Vero cells supplemented with 2% FBS and 125 µg/mL Trypsin using the Spearmann-Karber titer calculator. Significance was assessed by two-way ANOVA with Tukey's multiple comparison. * - < 0.05, ** - < 0.01, *** - < 0.001 and **** - < 0.0001.

UACC-62	NDV-GFP 24 hour	NDV-SIRPαFc 24 hour
NDV-αCD47 24 hour	0.038	0.007

Table S3. Significance values associated with multi-step growth curve in SKMEL-28 cells in Figure S2A. SKMEL-28 cells were infected, in triplicate, with NDV-GFP, NDV-αCD47 or NDV-SIRPα-Fc at a multiplicity of infection of 0.1 based on the number of cells seeded. A sample of the supernatant was harvested every 24 hours, for a total of 72 hours and virus concentration was determined by $TCID_{50}$ on Vero cells supplemented with 2% FBS and 125 µg/mL Trypsin using the Spearmann-Karber titer calculator. Significance was assessed by two-way ANOVA with Tukey's multiple comparison. * - < 0.05, ** - < 0.01, *** - < 0.001 and **** - < 0.0001.

**SKMEL-28**	**NDV-GFP 24 hour**	**NDV-SIRPα-Fc 24 hour**
NDV-αCD47 24 hour	N/A	< 0.001
NDV-GFP 24 hour	N/A	0.014

Table S4. Significance values associated with B16-F10 resazurin assays in Figure S2B. The percent of metabolically active B16-F10 cells following infection with NDV-GFP, NDV-αCD47 or NDV-SIRPα-Fc at multiplicities of infection of 100, 10 and 1 relative to uninfected cells, in triplicate, at 24-, 48- and 72-hours post infection. Significance was assessed by two-way ANOVA with Tukey's multiple comparison. * - < 0.05, ** - < 0.01, *** - < 0.001 and **** - < 0.0001.

MOI	Time (hours)	Comparison	Significance
10	72	NDV-GFP vs NDV-SIRPα-Fc	0.004
100	24	NDV-αCD47 vs NDV-SIRPα-Fc	0.046
		NDV-αCD47 vs NDV-GFP	0.048

Table S5. Significance values associated with UACC-62 resazurin assays in Figure S2B. The percent of metabolically active UACC-62 cells following infection with NDV-GFP, NDV-αCD47 or NDV-SIRPα-Fc at multiplicities of infection of 100, 10 and 1 relative to uninfected cells, in triplicate, at 24-, 48- and 72-hours post infection. Significance was assessed by two-way ANOVA with Tukey's multiple comparison. * - < 0.05, ** - < 0.01, *** - < 0.001 and **** - < 0.0001.

MOI	Time (hours)	Comparison	Significance
1	24	NDV-αCD47 vs NDV-SIRPα-Fc	< 0.001
		NDV-SIRPα-Fc vs NDV-GFP	< 0.001
	48	NDV-αCD47 vs NDV-SIRPα-Fc	< 0.001
		NDV-αCD47 vs NDV-GFP	0.004
10	24	NDV-αCD47 vs NDV-SIRPα-Fc	< 0.001
		NDV-αCD47 vs NDV-GFP	< 0.001
		NDV-GFP vs NDV-SIRPα-Fc	< 0.001
	72	NDV-αCD47 vs NDV-GFP	0.009
100	24	NDV-αCD47 vs NDV-SIRPα-Fc	< 0.001
		NDV-αCD47 vs NDV-GFP	< 0.001
	72	NDV-αCD47 vs NDV-GFP	0.044

Table S6. Significance values associated with SKMEL-28 resazurin assays in Figure S2B. The percent of metabolically active SKMEL-28 cells following infection with NDV-GFP, NDV-αCD47 or NDV-SIRPα-Fc at multiplicities of infection of 100, 10 and 1 relative to uninfected cells, in triplicate, at 24-, 48- and 72-hours post infection. Significance was assessed by two-way ANOVA with Tukey's multiple comparison. * - < 0.05, ** - < 0.01, *** - < 0.001 and **** - < 0.0001.

MOI	Time (hours)	Comparison	Significance
1	48	NDV-αCD47 vs NDV-SIRPα-Fc	< 0.001
		NDV-αCD47 vs NDV-GFP	< 0.001
		NDV-GFP vs NDV-SIRPα-Fc	< 0.001
	72	NDV-αCD47 vs NDV-SIRPα-Fc	< 0.001
		NDV-αCD47 vs NDV-GFP	0.002
		NDV-GFP vs NDV-SIRPα-Fc	< 0.001
10	24	NDV-αCD47 vs NDV-SIRPα-Fc	0.003
		NDV-αCD47 vs NDV-GFP	< 0.001
	48	NDV-αCD47 vs NDV-SIRPα-Fc	< 0.001
		NDV-GFP vs NDV-SIRPα-Fc	< 0.001
	72	NDV-αCD47 vs NDV-SIRPα-Fc	0.02
		NDV-GFP vs NDV-SIRPα-Fc	0.004
100	24	NDV-αCD47 vs NDV-GFP	< 0.001
		NDV-GFP vs NDV-SIRPα-Fc	< 0.001
	48	NDV-αCD47 vs NDV-SIRPα-Fc	< 0.001
		NDV-GFP vs NDV-SIRPα-Fc	< 0.001
	72	NDV-αCD47 vs NDV-SIRPα-Fc	0.015
		NDV-GFP vs NDV-SIRPα-Fc	< 0.001

Table S7. Antibodies used during flow cytometric staining. Each antibody was diluted as indicated and purchased from the corresponding supplier. hIgG – human immunoglobulin G, BV – Brilliant Violet.

Antibody	Dilution	Supplier	Catalogue #
PE-Anti-CD47	1/200	BioLegend	127507
7-AAD	1/10	BioLegend	420403
AlexaFluor647-Anti-Calreticulin	1/200	Abcam	Ab196159
Anti-CD16/32	1/200	BioLegend	101329
PE-Cy7-Anti-hIgG	1/200	BioLegend	366907
PE-Anti-F4/80	1/200	BioLegend	123110
PE-Cy7-Anti-CD11c	1/400	BioLegend	117317
APC-Anti-SIRPα	1/200	BioLegend	144013
APC-Cy7-Anti-CD45.2	1/200	BioLegend	109830
APC-Cy7-Anti-MHCII	1/200	BioLegend	107627
PE-Fire810-Anti-MHCII	1/200	BioLegend	107667
BV421-Anti-XCR1	1/200	BioLegend	148216
BV510-Anti-B220	1/200	BioLegend	103247
BV605-Anti-CD80	1/200	ThermoFisher	406-0801-80
APC-Cy7-Anti-CD86	1/200	BioLegend	159217
SparkRed718-Anti-PD-L1	1/200	BioLegend	124348
PE-Anti-Thy1.1	1/200	BioLegend	202523
PerCP-Anti-CD3	1/200	BioLegend	100325
BV650-Anti-CD8	1/200	BioLegend	100741
BV510-Anti-CD4	1/200	BioLegend	116025
FITC-Anti-NK1.1	1/200	BioLegend	108705
BV711-Anti-CD44	1/200	BioLegend	103057
BV785-Anti-PD-1	1/200	BioLegend	135225
BV605-Anti-CD62L	1/200	BioLegend	104437
